# Wittig reaction of cyclobisbiphenylenecarbonyl

**DOI:** 10.3762/bjoc.21.107

**Published:** 2025-07-14

**Authors:** Taito Moribe, Junichiro Hirano, Hideaki Takano, Hiroshi Shinokubo, Norihito Fukui

**Affiliations:** 1 Department of Molecular and Macromolecular Chemistry, Graduate School of Engineering, Nagoya University, Furo-cho, Chikusa-ku, Nagoya, Aichi 464-8603, Japan; 2 Institute for Advanced Research, Nagoya University, Furo-cho, Chikusa-ku, Nagoya, Aichi 464-8601, Japan; 3 Research Institute for Quantum and Chemical Innovation, Institutes of Innovation for Future Society and Integrated Research Consortium on Chemical Science (IRCCS), Nagoya University, Furo-cho, Chikusa-ku, Nagoya, 464-8603, Japan

**Keywords:** bathtub, chirality, cyclobisbiphenylenecarbonyl, figure-eight, Wittig reaction

## Abstract

Cyclobisbiphenylenecarbonyl (CBBC) represents a readily available chiral figure-eight macrocycle containing two carbonyl groups. However, the transformation of the carbonyl groups has been unexplored. Herein, we conducted the Wittig reaction of CBBC with methylenetriphenylphosphorane to furnish two chiral macrocycles containing one or two exocyclic olefin units. Owing to the transformation of carbonyl groups, the resulting products exhibit several unique physical and chemical properties: (1) the enhancement of configurational stability, (2) the appearance of fluorescence, and (3) the reductive carbon–carbon-bond formation between carbonyl and alkene units.

## Introduction

Figure-eight π-conjugated molecules represent chiral macrocycles with a twisted crossover structure [1–15]. Various figure-eight π-systems including aromatic hydrocarbons, belt-type extended π-systems, and porphyrinoids have been reported. The structural twisting in figure-eight macrocycles leads to cross-linked conjugation at the molecular center and a highly symmetric chiral structure with *D*_2_-symmetry. Consequently, figure-eight molecules often exhibit fascinating properties, such as unusual rearrangement reactions [9] and efficient circularly polarized luminescence (CPL) [10–12].

**Figure 1:**
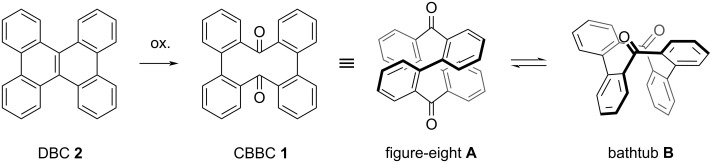
Synthesis and structures of CBBC **1**.

Cyclobisbiphenylenecarbonyl (CBBC) **1** is a figure-eight macrocycle, which is readily synthesized from commercially available dibenzo[*g*,*p*]chrysene (DBC, **2**) via oxidative inner-bond cleavage (Figure 1) [16–17]. CBBC **1** was first synthesized by Suszko and Schillak in 1934 using sodium dichromate as an oxidant [16]. Recently, our group developed a scalable, catalytic, and enantioselective protocol to furnish CBBC **1** [17]. Several peripherally modified CBBC derivatives have also been prepared and were shown to have fascinating properties [17–21]. For example, carbazole-substituted donor–acceptor-type CBBC derivatives exhibit both efficient circularly polarized luminescence (CPL) and thermally activated delayed fluorescence (TADF), demonstrating that CBBC represents a promising building block for the design of advanced functional materials [17,21]. However, the transformation of the carbonyl groups in CBBC has been underexplored. Herein, we report the Wittig reaction of CBBC **1**. CBBC **1** undergoes structural change from a stable figure-eight conformation **A** to a metastable bathtub conformation **B** with a small energy difference of approximately 2 kcal mol^–1^ [21]. In this paper, we discuss the effect of the transformation of the carbonyl groups on the conformational change of the figure-eight structure. We thus intentionally depict flattened chemical structures in the reaction schemes.

## Results and Discussion

### Synthesis and characterization

**Scheme 1:**
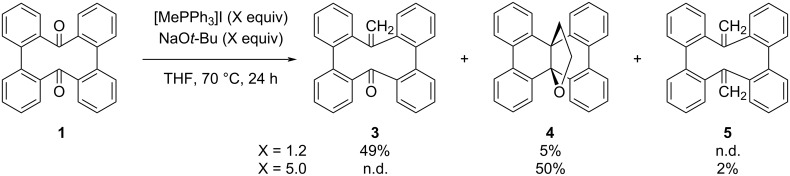
Wittig reactions of CBBC **1**.

Methylenetriphenylphosphorane was generated by mixing equimolar amounts of methyltriphenylphosphonium iodide ([MePPh_3_]I) and sodium *tert*-butoxide (NaO*t*-Bu) in THF. The Wittig reaction of CBBC **1** with 1.2 equiv of methylenetriphenylphosphorane afforded mono-olefin **3** in 49% yield as well as an internally functionalized dibenzo[*g*,*p*]chrysene (DBC) derivative **4** in 5% yield (Scheme 1). The use of an excess amount of methylenetriphenylphosphorane (5.0 equiv) afforded compound **4** in a higher yield of 50%. In addition, the reaction furnished bis-olefin **5** in 2% isolated yield which is lower than the estimated yield by ^1^H NMR measurement of the crude mixture (11%). This is due to the partial loss of the product during purification to remove a trace amount of DBC **2**, which was generated as a byproduct and exhibited similar polarity as compound **5**. The obtained compounds **3**, **4**, and **5** were identified using nuclear magnetic resonance (NMR) spectroscopy and mass spectrometry (MS) (see Supporting Information File 1) as well as single crystal X-ray diffraction analysis (vide infra). Furthermore, the absence of carbonyl groups in bis-olefin **5** has been corroborated by Fourier transform infrared (FTIR) spectroscopy (Figure S16 in Supporting Information File 1).

Compound **4** could be generated through the reaction of compound **3** with phosphorus ylide. However, a reliable reaction mechanism remains unclear. A tentative mechanism that may be plausible is shown in Supporting Information File 1, Figure S21, which consists of (1) the nucleophilic attack of methylenetriphenylphosphorane to the *exo*-methylene group of **3**, (2) the intramolecular carbon–carbon-bond formation at the carbonyl group, and (3) the nucleophilic substitution of the thus generated alkoxide to form an oxygen-containing five-membered ring. At least, density functional theory (DFT) calculations support that the nucleophilic attack of methylenetriphenylphosphorane to the *exo*-methylene unit is slightly favorable over reaction with the carbonyl group (Figure S20, Supporting Information File 1), which will be due to the disrupted nucleophilic attack to the carbonyl group by the intramolecular steric repulsion toward the Bürgi–Dunitz angle. However, alternative mechanisms initiated by a conventional oxaphosphetane formation cannot not yet be ruled out.

**Figure 2:**
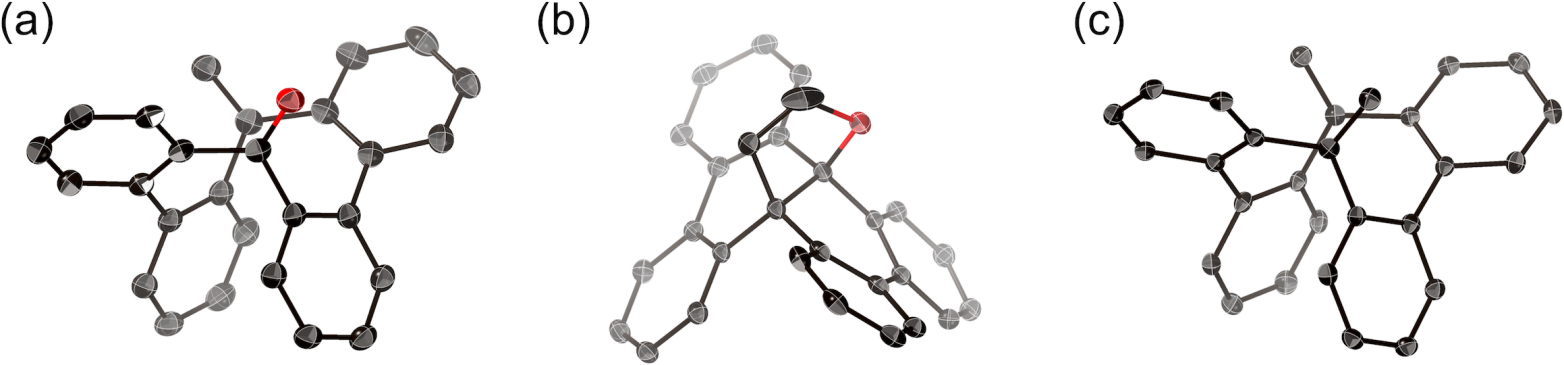
X-ray crystal structures of (a) **3**, (b) **4**, and (c) **5** with thermal ellipsoids at 50% probability; all hydrogen atoms are omitted for clarity.

The structures of compounds **3**, **4**, and **5** were determined by X-ray diffraction analysis (Figure 2). Mono-olefin **3** and bis-olefin **5** adopt a bathtub-like chiral macrocyclic structure rather than figure-eight conformation. Both compounds crystallize as a racemic pair of enantiomers with *P*2_1_/*c* and *Cc* space groups, respectively. The bond lengths at the exocyclic olefin units of **3** and **5** are 1.336(2) and 1.333(3)–1.337(3) Å, respectively, which are typical for carbon–carbon double bonds. The (CH_2_CH_2_O)-substituted DBC derivative **4** adopts a double-helicene-like structure similarly to other internally functionalized DBC derivatives [22]. The dihedral angle between the mean planes of the two terminal benzene units is 83°, which is comparable to those of other derivatives.

**Figure 3:**
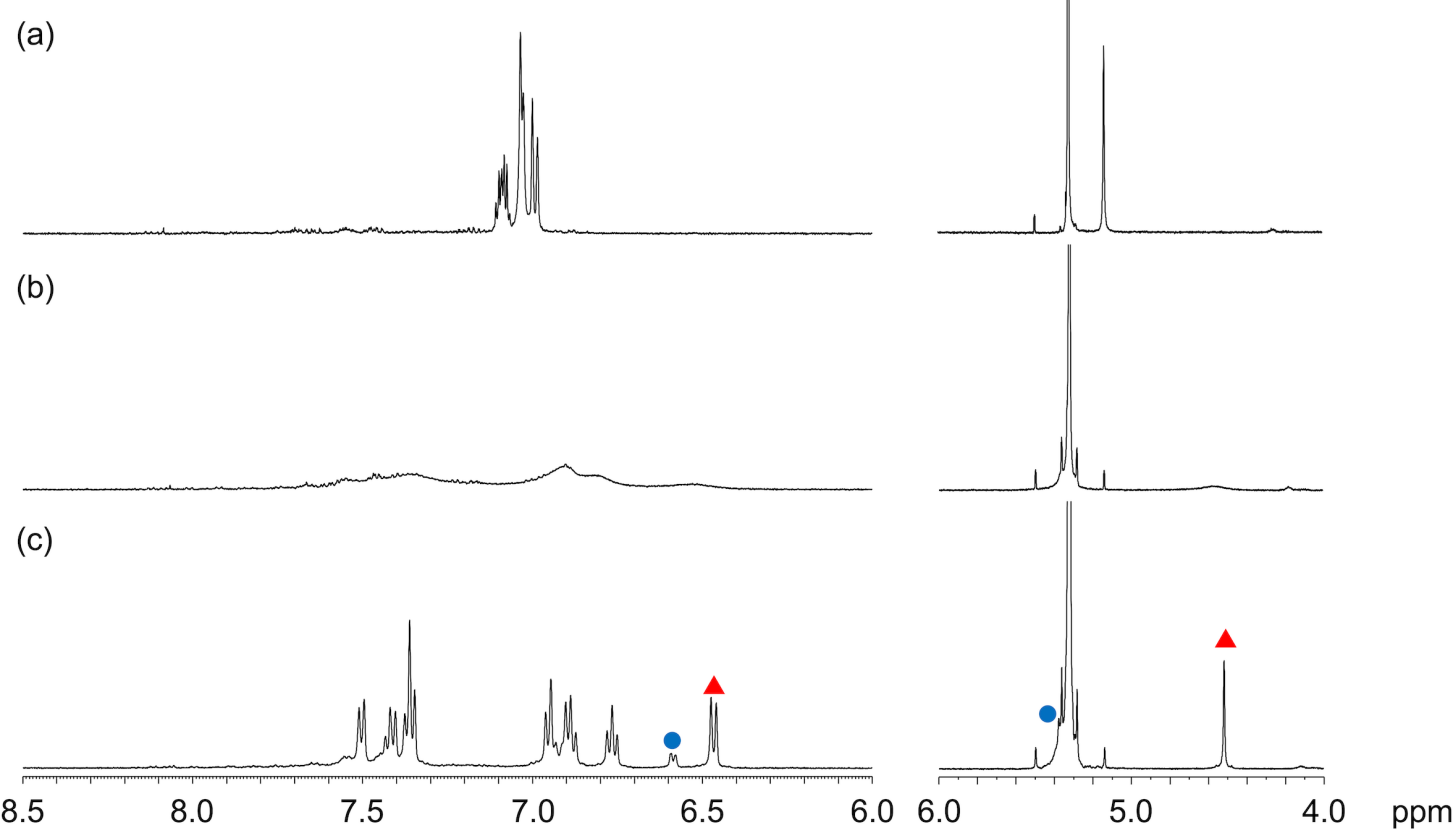
VT ^1^H NMR spectra of **5** in CD_2_Cl_2_ at (a) 298 K, (b) 243 K, and (c) 203 K. Blue circle and red triangle mean selected signals due to figure-eight and bathtub conformations, respectively.

Next, products **3** and **5** were analyzed by variable temperature (VT) ^1^H NMR spectroscopy. The ^1^H NMR spectrum of bis-olefin **5** in CD_2_Cl_2_ at 298 K shows a symmetric pattern, in which the signal due to the methylene protons appears as one singlet (Figure 3). The decrease of temperature to 243 K resulted in the broadening of the ^1^H NMR spectrum and the appearance of two sets of signals which sharpened upon further decrease of the temperature. These are attributable to the mixture of conformers with the figure-eight conformation as minor and the bathtub conformation as major conformer with a ratio of ca. 1:7. The obtained temperature-dependent ^1^H NMR data were subjected to the van't Hoff plot, affording an enthalpy Δ*H* and an entropy Δ*S* of 1.5 kcal mol^−1^ and 3.2 cal K^−1^ mol^−1^, respectively (Figure S26 in Supporting Information File 1). These physical parameters give a free energy Δ*G*_298_ of 0.55 kcal mol^−1^, indicating approximately a 2:5 ratio of figure-eight and bathtub conformations at room temperature. Mono-olefin **3** exhibited similar temperature-dependent ^1^H NMR changes, which furnished Δ*H* of 3.1 kcal mol^−1^ and Δ*S* of 11 cal K^−1^ mol^−1^ for the (figure-eight)–bathtub interconversion (Figure S25 in Supporting Information File 1). These parameters afforded Δ*G*_298_ of −0.22 kcal mol^−1^, indicating that the figure-eight conformation is slightly preferred at room temperature with approximately a 3:2 ratio of figure-eight and bathtub conformations. We have also estimated the activation barriers of the interconversion of **3** and **5** between the figure-eight and bathtub conformations by measuring VT ^1^H NMR spectra in toluene-*d*_8_ because the signals due to the *exo*-methylene groups overlapped with the solvent signal in CD_2_Cl_2_. The thus obtained activation barriers of **3** and **5** were 11 and 12 kcal mol^–1^ at 263 and 253 K, respectively (Figure S27 in Supporting Information File 1).

Previous DFT calculations at the B3LYP/6-31G(d) level of theory suggested that the bathtub conformation of CBBC **1** is slightly unfavorable than the figure-eight conformation by 2.4 kcal mol^−1^ [21]. On the other hand, the current DFT calculations suggest that the bathtub conformation of bis-olefin **5** is rather favorable by 0.3 kcal mol^−1^, which is in accordance with the temperature-dependent ^1^H NMR measurements. The relatively preferable formation of bathtub conformation is attributable to the destabilization of the figure-eight structures by the intramolecular steric repulsion between the *exo*-methylene units and neighboring benzene rings.

### Resolution

The resolution of rac-**3** and rac-**5** was conducted using high-performance liquid chromatography (HPLC) equipped with DAICEL CHIRALPAK IE as the chiral stationary phase (eluent: CH_2_Cl_2_/hexane 3:2 for **3** and 1:9 for **5**). The absolute configurations of the enantiomers were determined by transformation of enantiomerically pure CBBC (*P*,*P*)-**1**, whose configuration was previously confirmed [17]. The (*P*,*P*)-figure-eight conformation of CBBC **1** corresponds to the (*R*_a_,*R*_a_)-bathtub conformation, whose configuration is based on the axial chirality of the biaryl segment. Consequently, the 1st fractions of **3** and **5** were determined to be (*S*_a_,*S*_a_) and (*R*_a_,*R*_a_), respectively (see Supporting Information File 1, Figures S1 and S2).

The resolution of (CH_2_CH_2_O)-substituted DBC derivative **4** at ambient temperature was examined using DAICEL CHIRALPAK IA–IE (eluent: CH_2_Cl_2_/hexane and 2-propanol/hexane). However, the resolution was unsuccessful due to the low racemization barrier as with structurally similar methylenedioxy-substituted DBC derivative [22].

### Racemization dynamics

The racemization barriers of CBBC **1**, mono-olefin **3**, and bis-olefin **5** were evaluated by monitoring the decrease of circular dichroism (CD) signals in 1,2-dichlorobenzene at 170 °C (Supporting Information File 1, Figures S22–S24). The decrease of CD intensity was fitted by a single exponential curve, affording half-lifes of 1.3 h for **1**, 6.4 h for **3**, and 29 h for **5**. These results indicate that the transformation of carbonyl groups to exocyclic olefins is effective to retard racemization.

**Figure 4:**
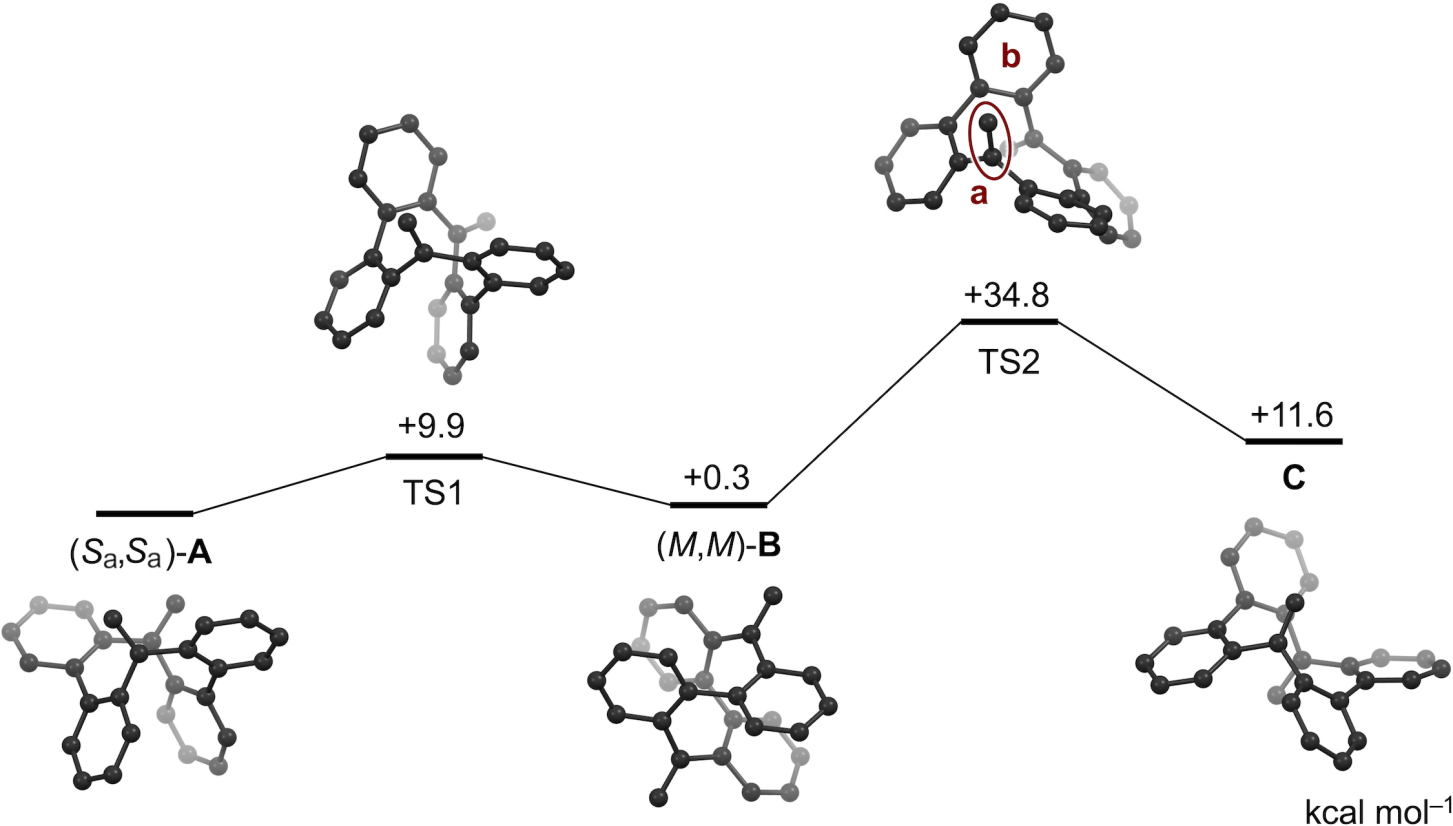
Simulated dynamics of bis-olefin **5** at the B3LYP/6-31G(d) level of theory. The description for the configuration of **A** and **B** are based on the helical chirality of the 1,1-diphenylethylene units and the axial chirality of the biaryl segments, respectively.

The racemization dynamics of **5** was investigated by DFT calculations at the B3LYP/6-31G(d) level of theory, employing the Gaussian 16 software package and the global reaction route mapping (GRRM17) [23] program (Figure 4). The interconversion between figure-eight conformation (*M*,*M*)-**B** and bathtub conformation (*S*_a_,*S*_a_)-**A** is feasible with a small activation barrier of 9.9 kcal mol^−1^. The figure-eight conformer (*M*,*M*)-**B** untwists to adopt an achiral conformation **C** with the *exo*-alkene units rotated inwards in opposite directions. These conformational changes are almost identical to those of CBBC **1**. However, the racemization barrier of **5** (34.8 kcal mol^−1^) is larger than that of CBBC **1** (33.7 kcal mol^−1^), which accords with the experimental results. In the transition state TS2, the exocyclic olefin unit **a** is close to the adjacent benzene ring **b**, which causes intramolecular steric repulsion to increase the racemization barrier.

### Optical and chiroptical properties

**Figure 5:**
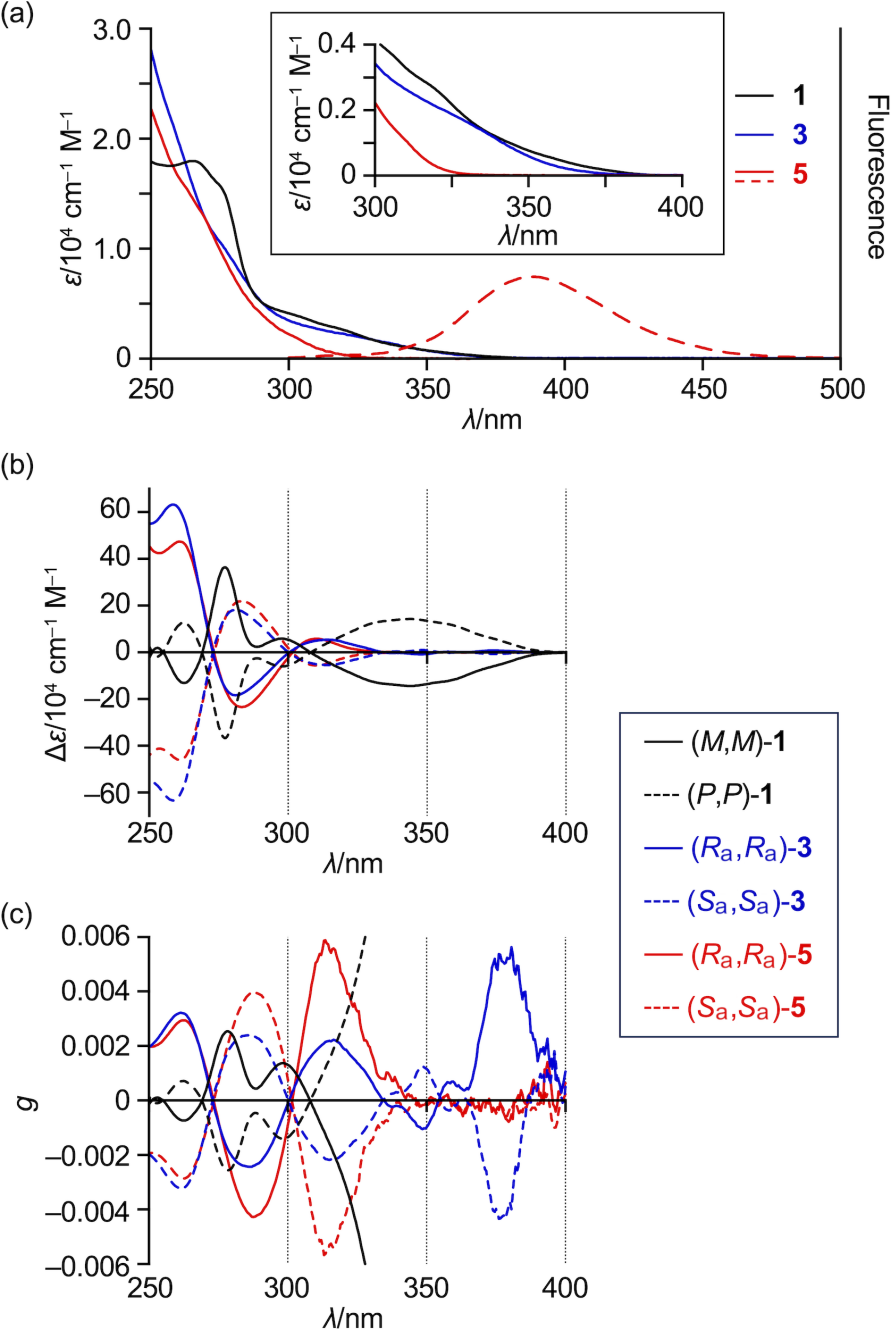
(a) UV–vis absorption (solid lines) and emission (dashed lines) spectra of **1** (black), **3** (blue), and **5** (red). (b) CD spectra of **1** (black), **3** (blue), and **5** (red). (c) CD *g* values of **1** (black), **3** (blue), and **5** (red). λ = wavelength; ε = extinction coefficient; solvent = CH_2_Cl_2_.

The UV–vis absorption spectra of CBBC **1**, mono-olefin **3**, and bis-olefin **5** are shown in Figure 5a. The absorption of **3** tails to 370 nm, which is comparable to the absorption end of CBBC **1**. On the other hand, the absorption of bis-olefin **5** is blue-shifted, tailing to 325 nm. In the case of CBBC **1**, the contribution of n–π* transition due to the carbonyl groups affords weak absorption in the 300–380 nm range [17]. Consequently, the blue-shifted absorption of **5** compared to those of **1** and **3** could result from the loss of carbonyl groups. The relatively large optical HOMO–LUMO gap of **5** despite the presence of 26 sp^2^ carbons is due to the cross-conjugation at the exocyclic olefins. Mono-olefin **3** is virtually non-emissive, similarly to CBBC **1**, which could originate from the non-radiative decay via intersystem crossing due to the carbonyl group. In sharp contrast, bis-olefin **5** fluoresces at 389 nm with a quantum yield of 7.5% and a lifetime of 6.0 ns. The radiative and non-radiative decay rate constants are calculated to be 1.3 × 10^7^ s^−1^ and 1.5 × 10^8^ s^−1^, respectively.

The CD spectra and the dissymmetry factors (*g*) of enantiomers of CBBC **1**, mono-olefin **3**, and bis-olefin **5** are shown in Figure 5b and Figure 5c, respectively. These spectra are observed as mirror images for enantiomers. The shapes of the CD spectra of mono-olefin **3** and bis-olefin **5** are essentially identical except for nearly forbidden transitions of **3** in the 340–400 nm range. While the maximum *g* value of CBBC **1** is approximately 0.03, the *g* values of mono-olefin **3** and bis-olefin **5** are lower than 0.006. We conducted TD-DFT calculations for both the bathtub and figure-eight conformations of compounds **3** and **5**, indicating that the signs of CD signals are reversal in most spectral range (Figure S18 and Figure S19 in Supporting Information File 1). Consequently, the low *g* values of **3** and **5** are attributable to the offset of CD signals due to the coexistence of two conformations.

### Reactivity

**Scheme 2:**
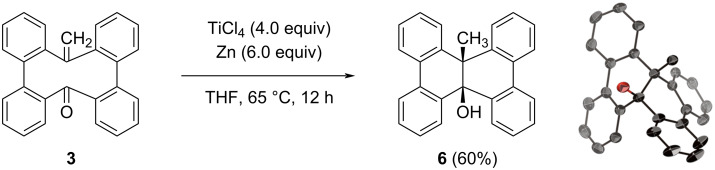
Conversion of mono-olefin **3** to internally functionalized DBC derivative **6**.

The reactivity of the Wittig products was examined. Mono-olefin **3** was treated with TiCl_4_ and zinc powder in THF at 65 °C, which are typical conditions for the McMurry coupling (Scheme 2) [24–25]. This reaction afforded an internally functionalized DBC derivative **6** in 60% yield, which adopts an unsymmetric structure with methyl and hydroxy groups on the central carbon atoms. The structure of compound **6** has been confirmed by X-ray diffraction analysis. On the other hand, the treatment of bis-olefin **5** under the same conditions recovered the starting material, which highlights the distinctive role of the carbonyl group for the reductive carbon–carbon-bond formation from **3**.

## Conclusion

The Wittig reaction of CBBC **1** with methylenetriphenylphosphorane furnished two (exocyclic olefin)-containing macrocycles **3** and **5** as well as an internally functionalized DBC derivative **4**. Compounds **3** and **5** adopt a bathtub-like conformation in the solid state. In solution, both figure-eight and bathtub conformations exist as an equilibrium mixture, in which the bathtub conformation is rather preferable at low temperature. Mono-olefin **3** and bis-olefin **5** exhibit enhanced configurational persistency compared to CBBC **1**. Bis-olefin **5** fluoresces with a quantum yield of 7.5%, while CBBC **1** is non-emissive under ambient conditions. Mono-olefin **3** undergoes a reductive carbon–carbon-bond formation between carbonyl and alkene units upon treatment with TiCl_4_. The current study demonstrates that the transformation of the carbonyl groups of CBBC results in products with altered physical and chemical properties which may be beneficial for the development of advanced materials.

## Supporting Information

File 1 Experimental details and spectral data for all new compounds.

## Data Availability

All data that supports the findings of this study is available in the published article and/or the supporting information of this article.
