# Supplementary material for: Wittig reaction of cyclobisbiphenylenecarbonyl
Source: Beilstein J Org Chem. 2025 Jul 14;21:1454–61. doi: 10.3762/bjoc.21.107 (PMC12278106; doi:10.3762/bjoc.21.107)
Supplement: File 1 — Experimental details and spectral data for all new compounds. [file Beilstein_J_Org_Chem-21-1454-s001.pdf]

BEILSTEIN JOURNAL OF ORGANIC CHEMISTRY

Supporting Information

for

Wittig reaction of cyclobisbiphenylenecarbonyl

Taito Moribe, Junichiro Hirano, Hideaki Takano, Hiroshi Shinokubo and Norihito Fukui

Beilstein J. Org. Chem. **2025,** *21,* 1454–1461. doi:10.3762/bjoc.21.107

Experimental details and spectral data for all new compounds

License and Terms: This is a supporting information file under the terms of the Creative Commons Attribution License (https://creativecommons.org/licenses/by/4.0). Please note that the reuse, redistribution and reproduction in particular requires that the author(s) and source are credited and that individual graphics may be subject to special legal provisions.
The license is subject to the *Beilstein Journal of Organic Chemistry* terms and conditions: (https://www.beilstein-journals.org/bjoc/terms)

Table of contents

1. Instrumentation and materials S2

2. Experimental procedures and compound data S4

3. NMR spectra S9

4. Mass spectra S17

5. Crystal data S26

6. DFT calculations S28

7. Others S45

8. References S49

1. Instrumentation and materials

Materials: All used reagents, solvents, and materials were purchased from Tokyo Chemical Industry (TCI), FUJIFILM Wako Pure Chemical Corporation, Sigma-Aldrich, Kanto Chemical, Nakalai Tesque, BLDpharm, or Alfa Aesar. Unless otherwise noted, materials obtained from commercial suppliers were used without further purification. CBBC **1**[1] and [$MePPh_3$]I[2] were synthesized according to the literature.[1] Dry THF was supplied from the Glass Countor solvent purification system. $TiCl_4$ was distilled before use. If not mentioned otherwise, all reactions were performed under standard conditions (at room temperature, at ambient pressure, and under air).

Chromatography: Analytical thin layer chromatography was performed using fluorescent-labeled silica-coated plates (TLC silica gel 60 F_{254}, Merck). Detection was conducted using UV light (λ_{ex} = 254 nm). Flash column chromatography was performed using silica gel (Wakogel 60N (particle size: 63–212 μm), C-300 (particle size: 45–75 μm), or C-400HG (particle size: 20–40 μm)). The preparative resolution was conducted using a JAI LabACE LC-7080 plus preparative HPLC with a chiral column (DAICEL CHIRALPAK IA, φ 20 mm × 250 mm). Analytical HPLC separation was performed on a Shimadzu CBM-20A system with a UV detector (SPD-20A), and Daicel Chiralpak IA-3 placed in a column oven (CTO-20AC).

Nuclear magnetic resonance (NMR) spectroscopy: ^{1}H NMR (500 MHz) and ^{13}C NMR (126 MHz) spectra were recorded on a Bruker AVANCE III HD spectrometer. Chemical shifts were reported as the delta scale in ppm relative to tetramethylsilane (δ = 0.00 ppm) and residual solvent signals (δ = 1.94 ppm for CD_3CN and 2.05 ppm for acetone-d_6) for ^{1}H NMR and $CDCl_3$ (δ = 77.16 ppm) and CD_3CN (δ = 1.32 ppm) for ^{13}C NMR. The following abbreviations were used to describe the multiplicities: s = singlet, d = doublet, t = triplet, m = multiplet.

Mass spectrometry (MS): High-resolution atmospheric pressure chemical ionization time-of-flight (APCI-TOF) and electrospray ionization time-of-flight (ESI-TOF) mass spectra were taken on a Bruker micrOTOF instrument using a positive ionization mode.

X-ray diffraction analysis: X-ray data were obtained using a Rigaku XtaLAB Synergy-R/NLN diffractometer with a MicroMax007HFMR X-ray generator and a HyPix-6000HE detector. The structures were solved using a direct method (SHELXT) and refined by a full-matrix least-squares method on F^2 for all reflections using the programs of SHELXL-2014. All nonhydrogen atoms were refined with anisotropic displacement parameters. The hydrogen atoms were placed in idealized positions and refined as riding models with the relative isotropic displacement parameters. Crystallographic data have been deposited with the Cambridge Crystallographic Data

Centre as a supplementary publication.

Spectroscopic measurements: UV–vis/NIR absorption spectra were recorded on a Shimadzu UV-2550 or JASCO V 670 spectrometer. Emission spectra were recorded on a JASCO FP-8550 spectrometer. Fluorescence lifetimes were recorded on a HAMAMATSU Quantaurus-Tau spectrometer. Circular dichroism (CD) spectra were recorded on a JASCO J-1500 CD spectrometer. Fourier transform infrared (FT-IR) spectra were recorded on a Shimadu IRSpirit-X spectrometer.

DFT calculations: All calculations were carried out using the *Gaussian 16* software package.[3] The structural optimization and frequency calculations were performed with the density functional theory (DFT) method with the restricted B3LYP[4] level, employing the 6-31G(d) basis sets. The TD-DFT calculations were conducted at the B3LYP/6-31G(d) level. Calculations for transition states were conducted using the *GRRM17*.[5] Geometry optimizations and vibration frequency calculations were performed at the B3LYP/6-31G level to confirm whether the stationary point structures are transition states.

2. Experimental procedures and compound data

Wittig reaction of CBBC 1 with 1.2 equiv of methylenetriphenylphosphorane

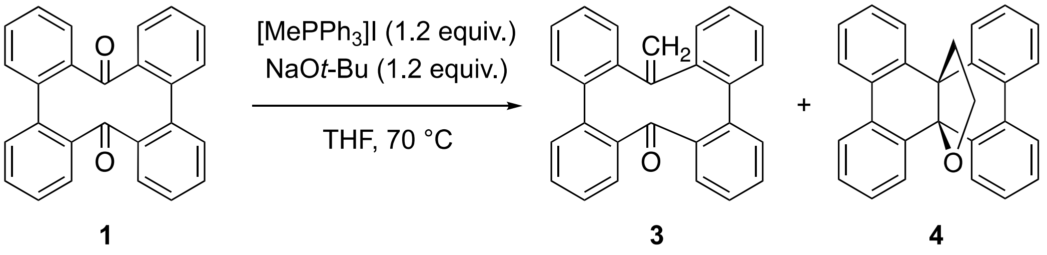

To a 50-mL Schlenk tube were added sodium *tert*-butoxide (0.16 g, 1.7 mmol) and methyltriphenylphosphonium iodide (0.67 g, 1.7 mmol) in an argon-filled glovebox. To the mixture, degassed dry THF (15 mL) was added. The mixture was stirred at 0 °C for 1 h. To the Schlenk tube was added CBBC **1** (0.50 g, 1.4 mmol). The mixture was stirred at 70 °C for 24 h. The reaction was quenched with HCl aq. (1 M). The organic materials were extracted with CH_2Cl_2. The organic extract was dried over anhydrous Na_2SO_4. After removal of the solvent in vacuo, the residue was separated by silica gel column chromatography (eluent: CH_2Cl_2/hexane 3:2), affording compound **3** (249 mg, 0.69 mmol, 49%) as a white solid and compound **4** (28 mg, 0.07 mmol, 5%) as a white solid.

Compound **3**: ^{1}H NMR (500 MHz, $CDCl_3$, 298 K): δ = 7.34 (dd, J_1 = 7.7 Hz, J_2 = 1.1 Hz, 2H), 7.23 (ddd, J_1 =7.6 Hz, J_2 = 7.6 Hz, J_3 = 1.4 Hz, 2H), 7.13–7.02 (m, 10H), 6.96 (dd, J_1 = 7.1 Hz, J_2 = 1.4 Hz, 2H), 5.33 (s, 2H) ppm; ^{13}C NMR (126 MHz, $CDCl_3$, 298 K): δ = 199.4, 148.6, 141.2, 139.8, 139.3, 138.7, 130.8, 130.4, 130.2, 129.4, 128.5, 127.7, 127.6, 127.1, 122.7 ppm; HRMS (APCI): $[M]^+$ Calcd for $C_{27}H_{18}O$ 358.1352; Found 358.1336; IR ν_{max} (neat)/cm^{-1} 3057(m), 1660(m), 1591(m), 1436(m), 1293(m), 925(m) 770(s), 747(s), 695(m), 638(s), 552(s), 517(m); The preparative resolution was conducted by recycling HPLC using a chiral column (Daicel Chiralpak IE: 20×250 mm, 254 nm UV detector, rt, eluent: CH_2Cl_2/hexane = 3/2, flow rate: 6 mL/min, injected substrate: 12 mg, obtained enantiomers: 4.7 mg as the 1st fraction and 3.7 mg as the 2nd fraction). Purities of the obtained enantiomers were determined by HPLC analysis using a chiral column (Daicel Chiralpak IE-3: 4.6×250 mm, 254 nm UV detector, 293 K, eluent: 60% CH_2Cl_2 in hexane, flow rate: 1 mL/min, retention time: 6.4 min for (*S*a,*S*a)-**3** and 8.3 min for (*R*a,*R*a)-**3**.

Compound **4**: ^{1}H NMR (500 MHz, acetone-d_6, 298 K): δ = 8.07 (dd, J_1 =7.9 Hz, J_2 = 1.4 Hz, 2H), 8.00–7.92 (m, 3H), 7.89 (dd, J_1 =7.9 Hz, J_2 = 1.2 Hz, 1H), 7.83 (dd, J_1 =7.8 Hz, J_2 = 1.3 Hz, 1H), 7.61–7.51 (m, 4H), 7.29 (ddd, J_1 =7.4 Hz, J_2 = 7.4 Hz, J_3 = 1.4 Hz, 1H), 7.19 (ddd, J_1 =7.3 Hz, J_2 = 7.3 Hz, J_3 = 1.4 Hz, 1H), 7.09–7.03 (m, 2H), 6.97 (dd, J_1 =7.6 Hz, J_2 = 1.1 Hz, 1H), 6.87 (dd, J_1 =7.9 Hz, J_2 = 1.0 Hz, 1H) 3.76 (ddd, J_1 =9.9 Hz, J_2 = 8.2 Hz, J_3 = 1.9 Hz, 1H), 3.52 (ddd, J_1 = 9.8 Hz, J_2 = 8.1 Hz, J_3 = 7.2 Hz, 1H), 2.99 (ddd, J_1 =12.4 Hz, J_2 = 6.7 Hz, J_3 = 1.7 Hz, 1H), 2.34 (ddd, J_1 =12.9 Hz, J_2 = 9.9 Hz, J_3 = 9.9 Hz, 1H) ppm; ^{13}C NMR (126 MHz, $CDCl_3$, 298K): δ = 137.5, 136.8, 135.6, 134.0, 133.4, 133.2, 132.7, 132.6, 130.2, 129.5, 129.1, 123.0, 128.6, 128.4, 128.1, 128.0, 127.8, 127.8, 127.5, 127.5, 125.1, 124.3, 124.1, 123.9, 84.1, 77.4, 65.8, 37.7 ppm; HRMS (APCI): $[M+H]^+$ Calcd for $C_{28}H_{21}O$ 373.1587; Found 373.1573; IR ν_{max} (neat)/cm^{-1} 2878(m), 1477(m), 1436(m), 1264(m), 1224(m), 1028(m), 988(m), 948(m), 758(s), 730(s), 552(m).

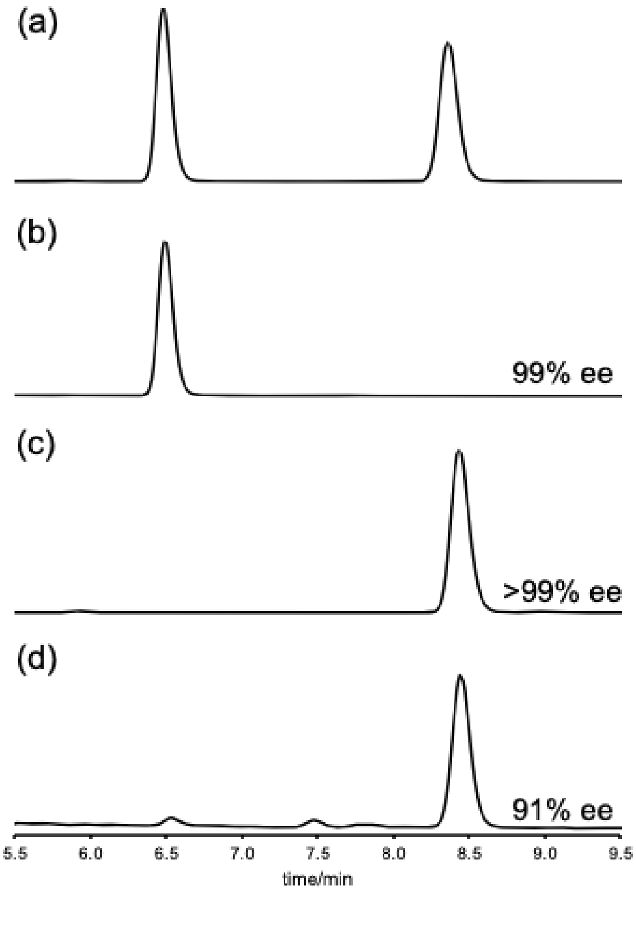

Figure S1. (a–c) HPLC charts of isolated (a) rac-**3**, (b) (S_a,S_a)-**3**, and (c) (R_a,R_a)-**3**. (d) HPLC chart of compound **3** synthesized from (*P*,*P*)-**1**.

Wittig reaction of CBBC 1 with 5.0 equiv of methylenetriphenylphosphorane

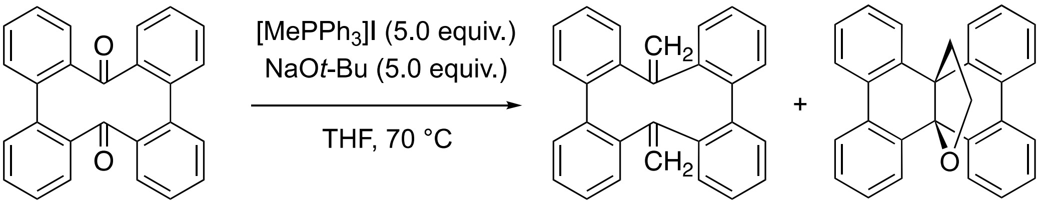

To a 50-mL Schlenk tube were added sodium *tert*-butoxide (0.67 g, 7.0 mmol) and methyltriphenylphosphonnium iodide (2.8 g, 7.0 mmol) in an argon-filled glovebox. To the mixture, degassed dry THF (15 mL) was added. The mixture was stirred at 0 °C for 1 h. To the Schlenk tube was added CBBC **1** (0.50 g, 1.4 mmol). The mixture was stirred at 70 °C for 24 h. The reaction was quenched with HCl aq. (1 M). The organic materials were extracted with CH_2Cl_2. The organic extract was dried over anhydrous Na_2SO_4. After removal of the solvent in vacuo, the residue was separated by silica gel column chromatography (eluent: CH_2Cl_2/hexane 1:9 to 6:4), affording compound **5** (11 mg, 0.031 mmol, 2%) as a white solid and compound **4** (263 mg, 0.70 mmol, 50%) as a white solid.

Compound **5**: ^{1}H NMR (500 MHz, $CDCl_3$, 298 K): δ = 7.11–6.99 (m, 16H), 5.16 (s, 4H) ppm; ^{13}C NMR (126 MHz, $CDCl_3$, 298K): δ = 149.6, 140.3, 140.2, 130.6, 128.9, 127.2, 127.0, 120.9 ppm; HRMS (APCI): $[M+H]^+$ Calcd for $C_{28}H_{21}$ 357.1638; Found 357.1647; IR ν_{max} (neat)/cm^{-1} 3051(m), 1614(m), 1471(m), 1436(m), 1321(m), 902(m), 764(s), 741(s), 609(m), 546(m), 523(m); The preparative resolution was conducted by recycling HPLC using a chiral column (Daicel Chiralpak IE: 20×250 mm, 254 nm UV detector, rt, eluent: CH_2Cl_2/hexane = 1/9, flow rate: 6 mL/min, injected substrate: 4.4 mg, obtained enantiomers: 1.9 mg as the 1st fraction and 2.3 mg as the 2nd fraction). Purities of the obtained enantiomers were determined by HPLC analysis using a chiral column (Daicel Chiralpak IC-3: 4.6×250 mm, 254 nm UV detector, 293 K, eluent: 10% CH_2Cl_2 in hexane, flow rate: 1 mL/min, retention time: 4.8 min for (*R*a,*R*a)-**5** and 5.5 min for (*S*a,*S*a)-**5**).

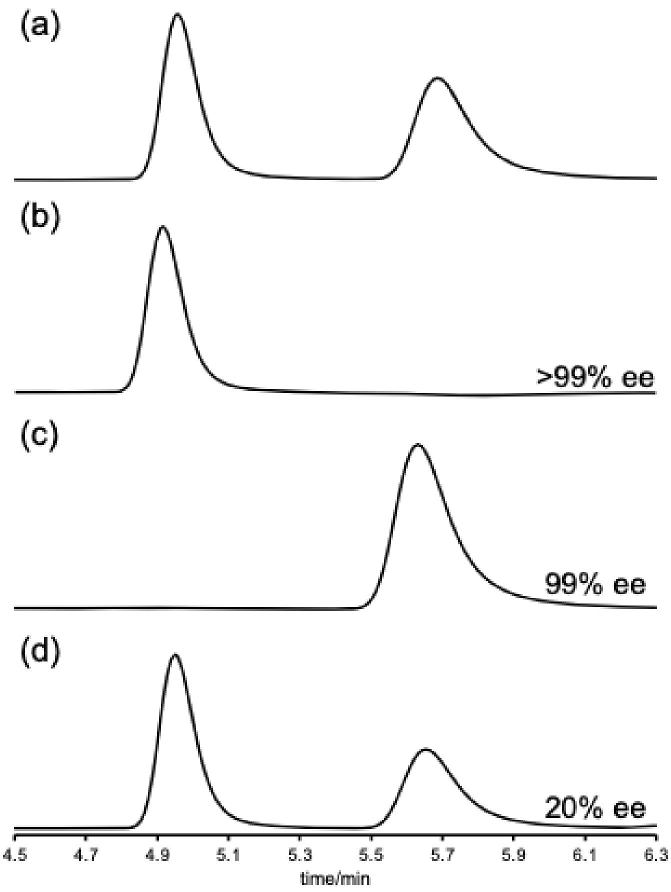

Figure S2. (a–c) HPLC charts of isolated (a) rac-**5**, (b) (R_a,R_a)-**5**, and (c) (S_a,S_a)-**5**. (d) HPLC chart of compound **5** synthesized from (*P*,*P*)-**1**.

Synthesis of compound 6

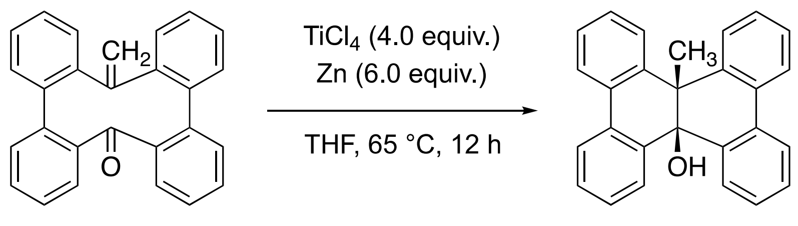

A Schlenk tube containing zinc powder (20 mg, 0.30 mmol) and compound **3** (18 mg, 0.050 mmol) was purged with N_2. To the mixture, degassed dry THF (1 mL) was added. After stirring at 0 °C, $TiCl_4$ (0.02 mL, 0.2 mmol) was added. The reaction mixture was stirred at 65 °C for 12 h. The reaction was quenched with $NaHCO_3$. The organic materials were extracted with

CH_2Cl_2. The organic extract was dried over anhydrous Na_2SO_4. After removal of the solvent in vacuo, the residue was separated by silica gel column chromatography (eluent: CH_2Cl_2/hexane 1:1), affording compound **6** (11 mg, 0.030 mmol, 60%) as a white solid.

Compound **6**: ^{1}H NMR (500 MHz, $CDCl_3$, 298 K): δ = 7.99 (dd, J_1 = 6.8 Hz, J_2 = 1.8 Hz, 1H), 7.83 (ddd, J_1 = 7.3 Hz, J_2 = 4.6 Hz, J_3 = 2.1 Hz, 1H), 7.79–7.75 (m, 2H), 7.63 (dd, J_1 = 7.9 Hz, J_2 = 1.1 Hz, 1H), 7.56 (dd, J_1 = 7.8 Hz, J_2 = 1.2 Hz, 1H), 7.54–7.47 (m, 4H), 7.16 (ddd, J_1 = 7.7 Hz, J_2 = 7.7 Hz, J_3 = 1.4, 1H), 7.05 (ddd, J_1 = 7.5 Hz, J_2 = 7.5 Hz, J_3 = 1.3, 1H), 6.91 (ddd, J_1 = 7.4 Hz, J_2 = 7.4 Hz, J_3 = 1.3, 1H), 6.87 (ddd, J_1 = 7.7 Hz, J_2 = 7.7 Hz, J_3 = 1.3, 1H), 6.61 (dd, J_1 = 7.8 Hz, J_2 = 1.2 Hz, 1H), 6.52 (dd, J_1 = 7.8 Hz, J_2 = 1.1 Hz, 1H), 2.22 (s, 1H), 1.58 (s, 3H) ppm; ^{13}C NMR (126 MHz, CD_3CN, 298 K): δ = 143.8, 140.4 140.0, 140.0, 134.8, 134.0, 133.7, 133.5, 129.7, 129.3, 128.7, 128.6, 128.5, 128.5, 128.2, 128.0, 127.8, 126.3, 125.3, 124.5, 124.0, 123.8, 77.3, 48.8, 19.8 ppm (Two peaks are missing due to the overlapping.); HRMS (APCI): $[M]^+$ Calcd for $C_{27}H_{20}O$ 360.1509; Found 360.1513; IR ν_{max} (neat)/cm^{-1} 3459(m), 3062(m), 1482(m), 1442(m), 1362(m), 1195(m), 1017(m), 758(s), 735(s), 643(m), 580(m), 506(m), 442(m).

3. NMR spectra

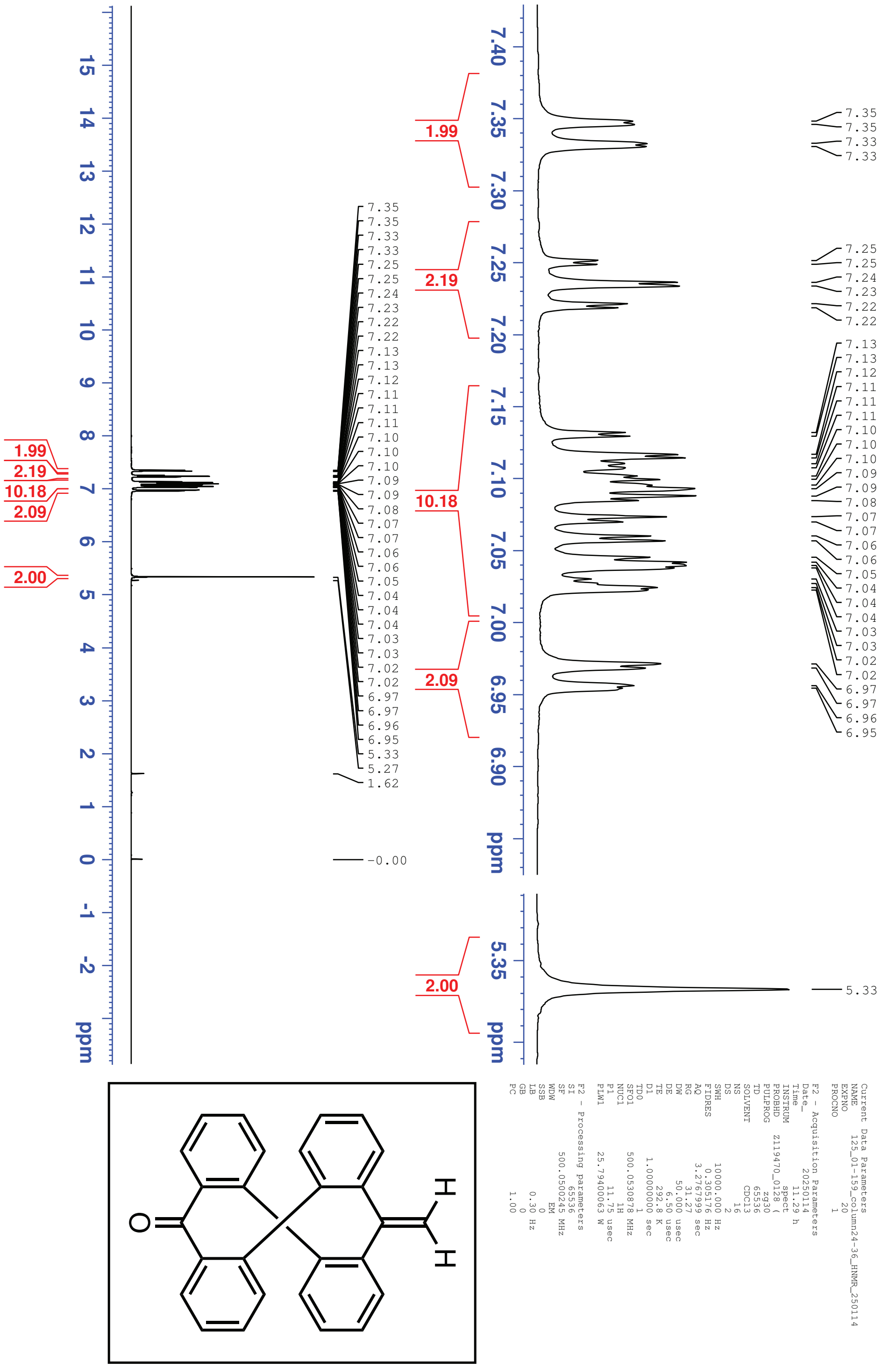

Figure S1. ^{1}H NMR spectrum of **3** in $CDCl_3$ at 25 °C.

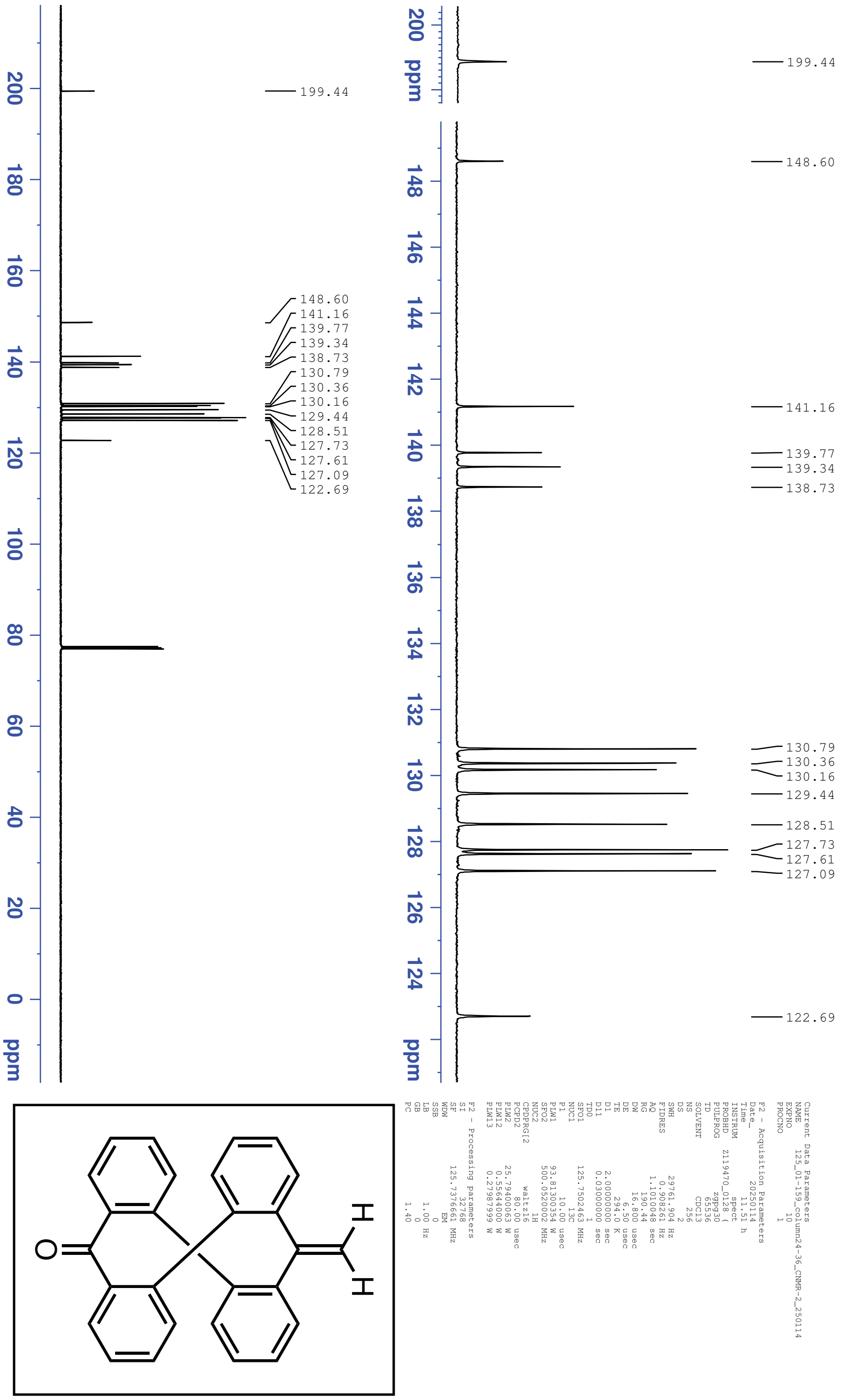

Figure S2. ^{13}C NMR spectrum of **3** in $CDCl_3$ at 25 °C.

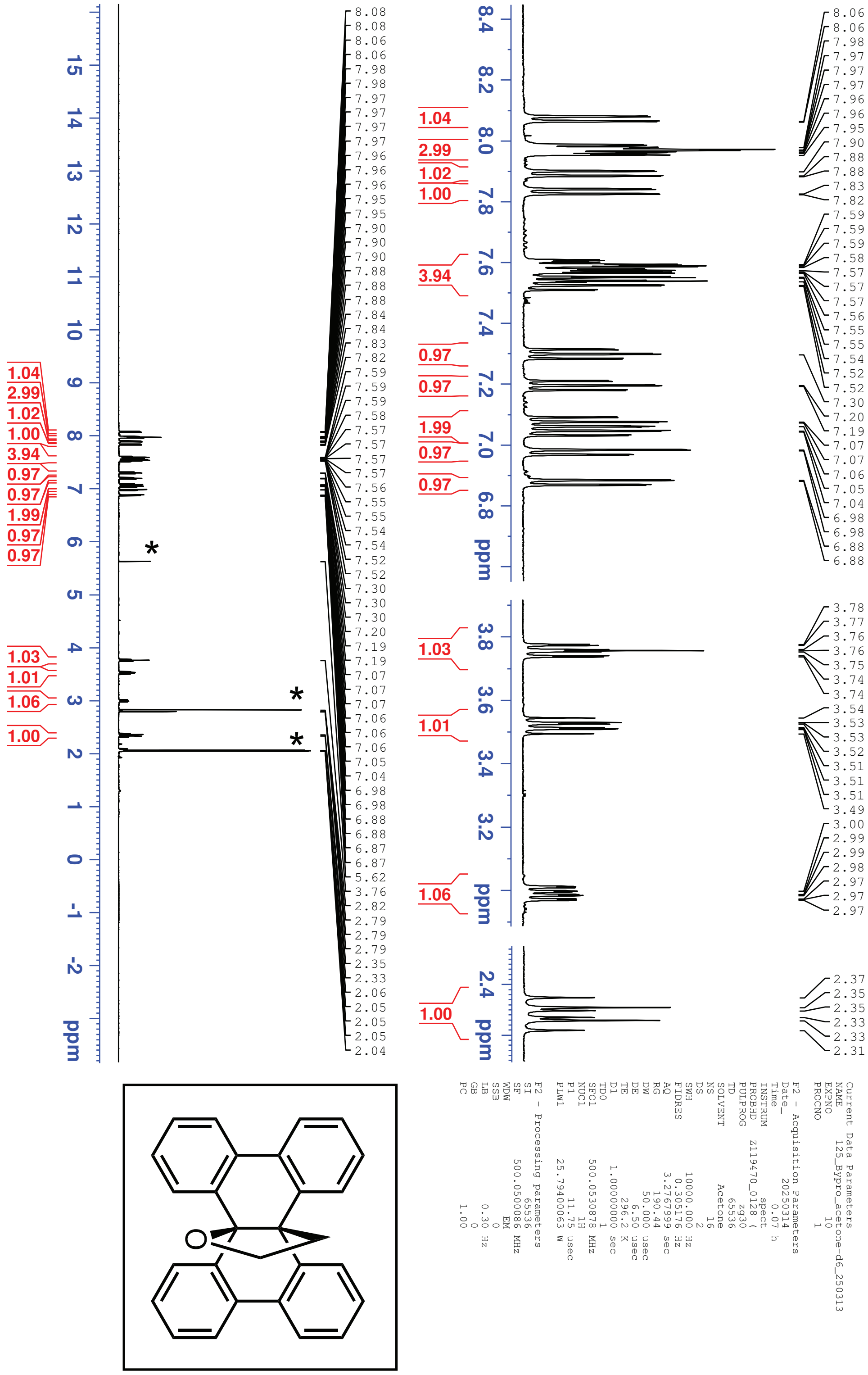

Figure S3. ^{1}H NMR spectrum of **4** in acetone-d_6 at 25 °C.

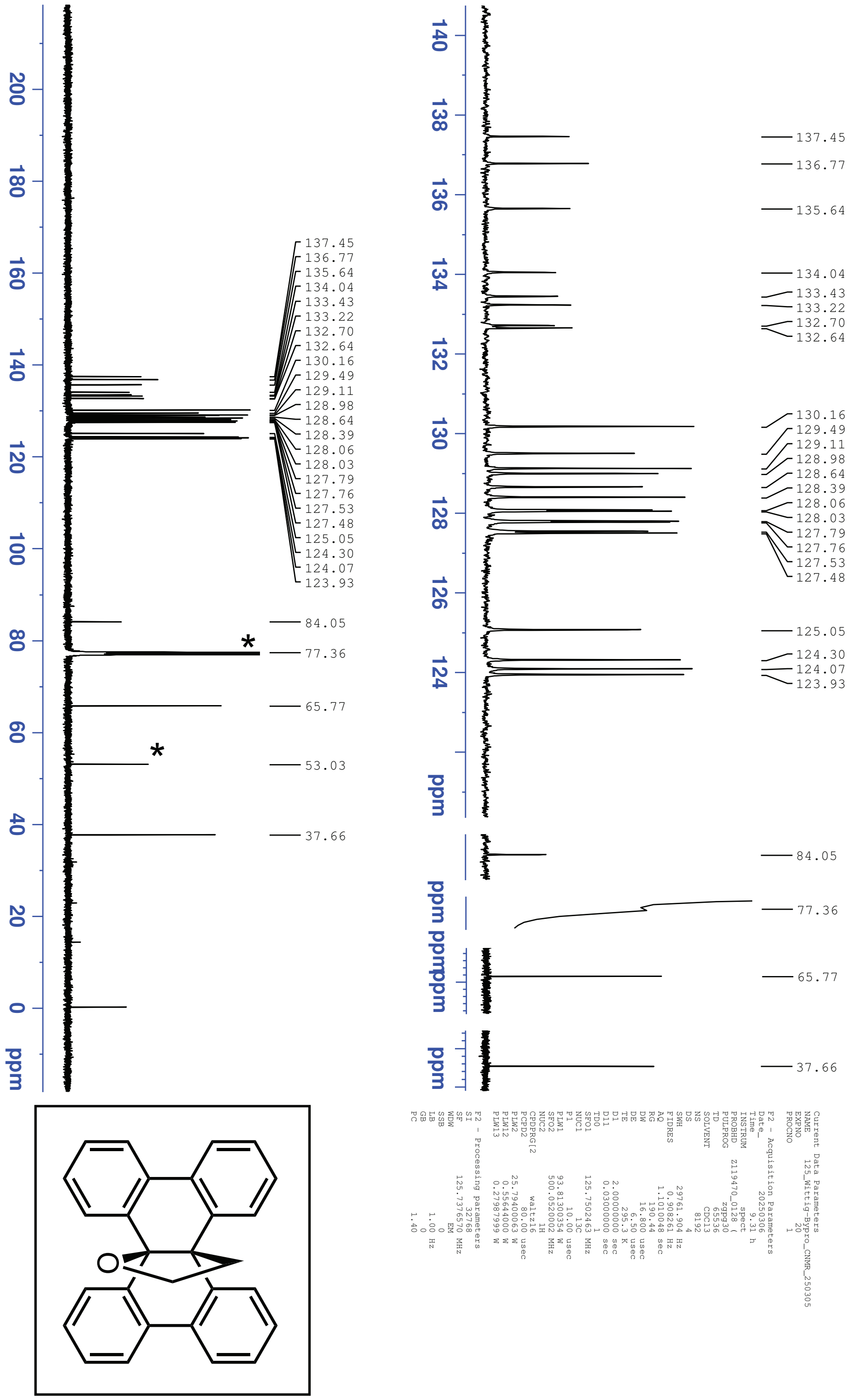

Figure S4. ^{13}C NMR spectrum of **4** in $CDCl_3$ at 25 °C.

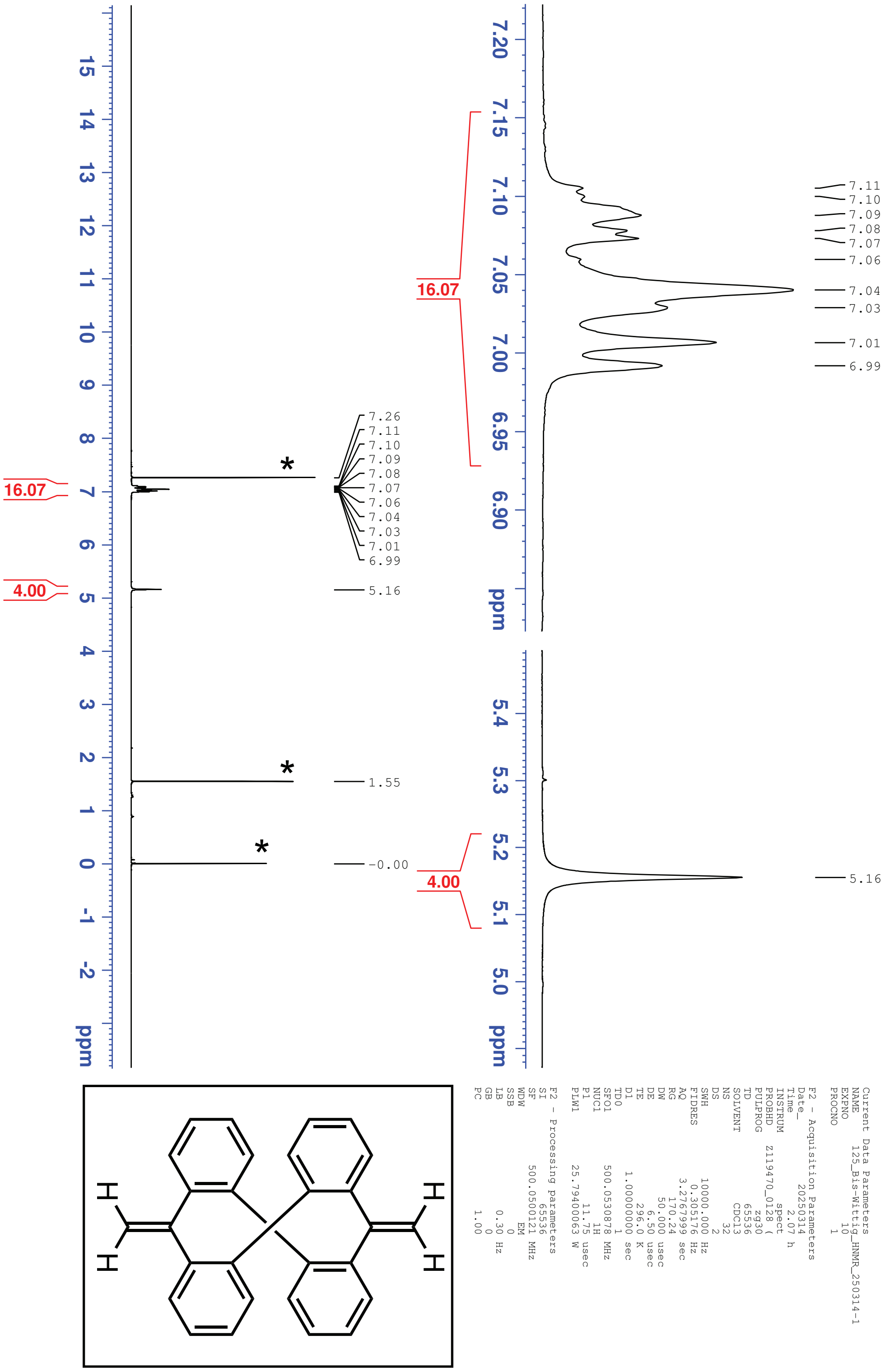

Figure S5. ^{1}H NMR spectrum of **5** in $CDCl_3$ at 25 °C.

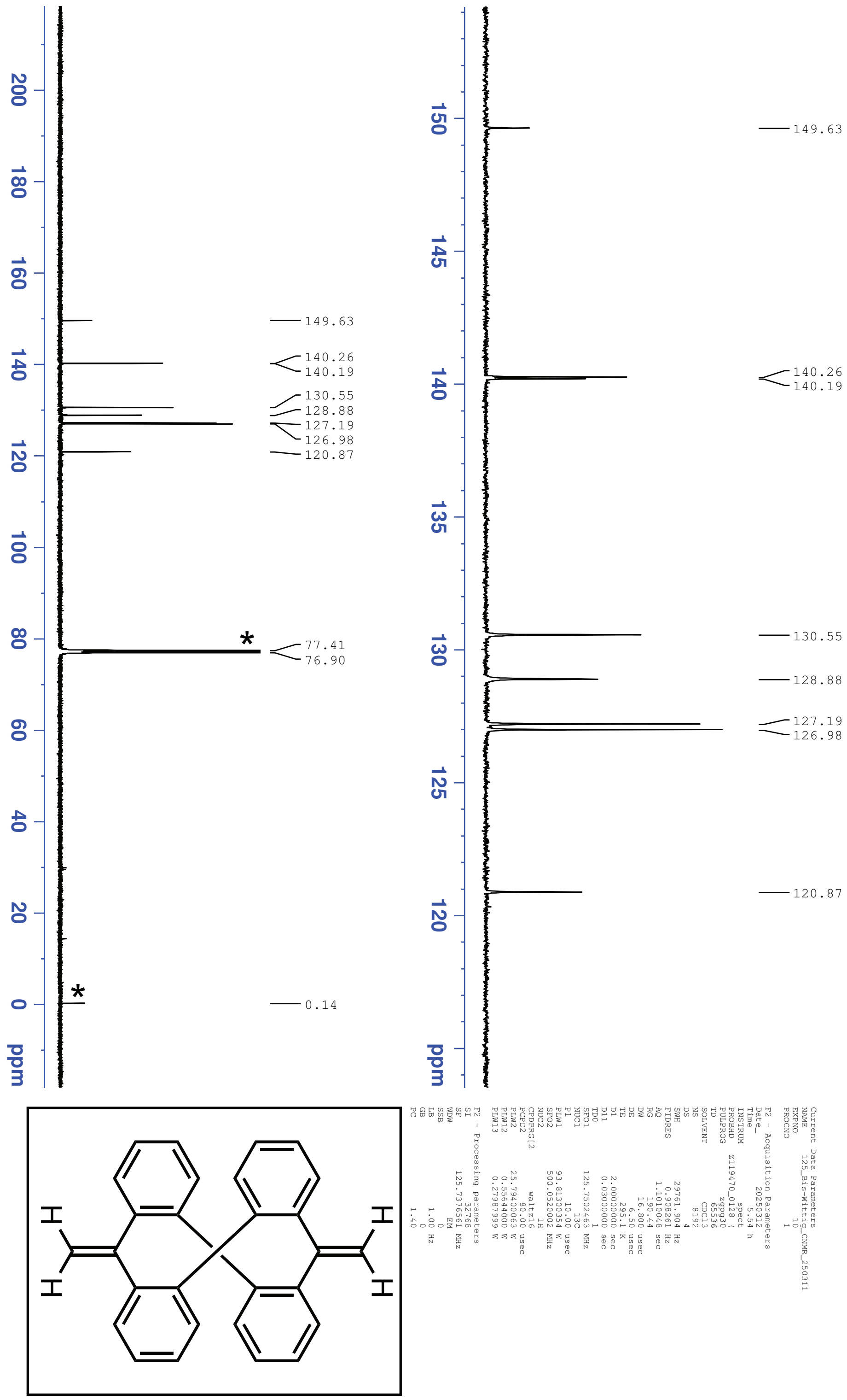

Figure S6. ^{13}C NMR spectrum of **5** in $CDCl_3$ at 25 °C.

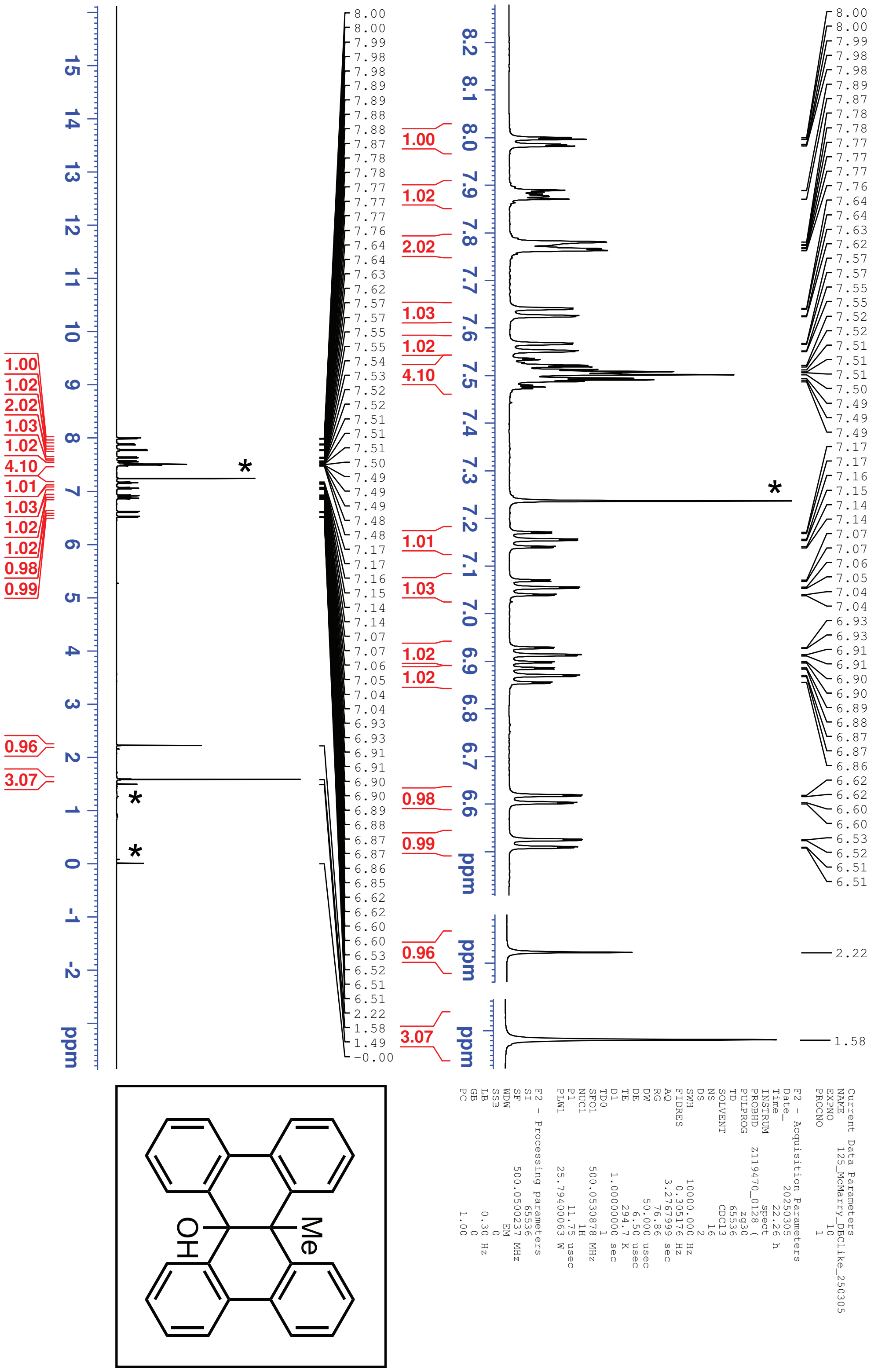

Figure S7. ^{1}H NMR spectrum of **6** in $CDCl_3$ at 25 °C.

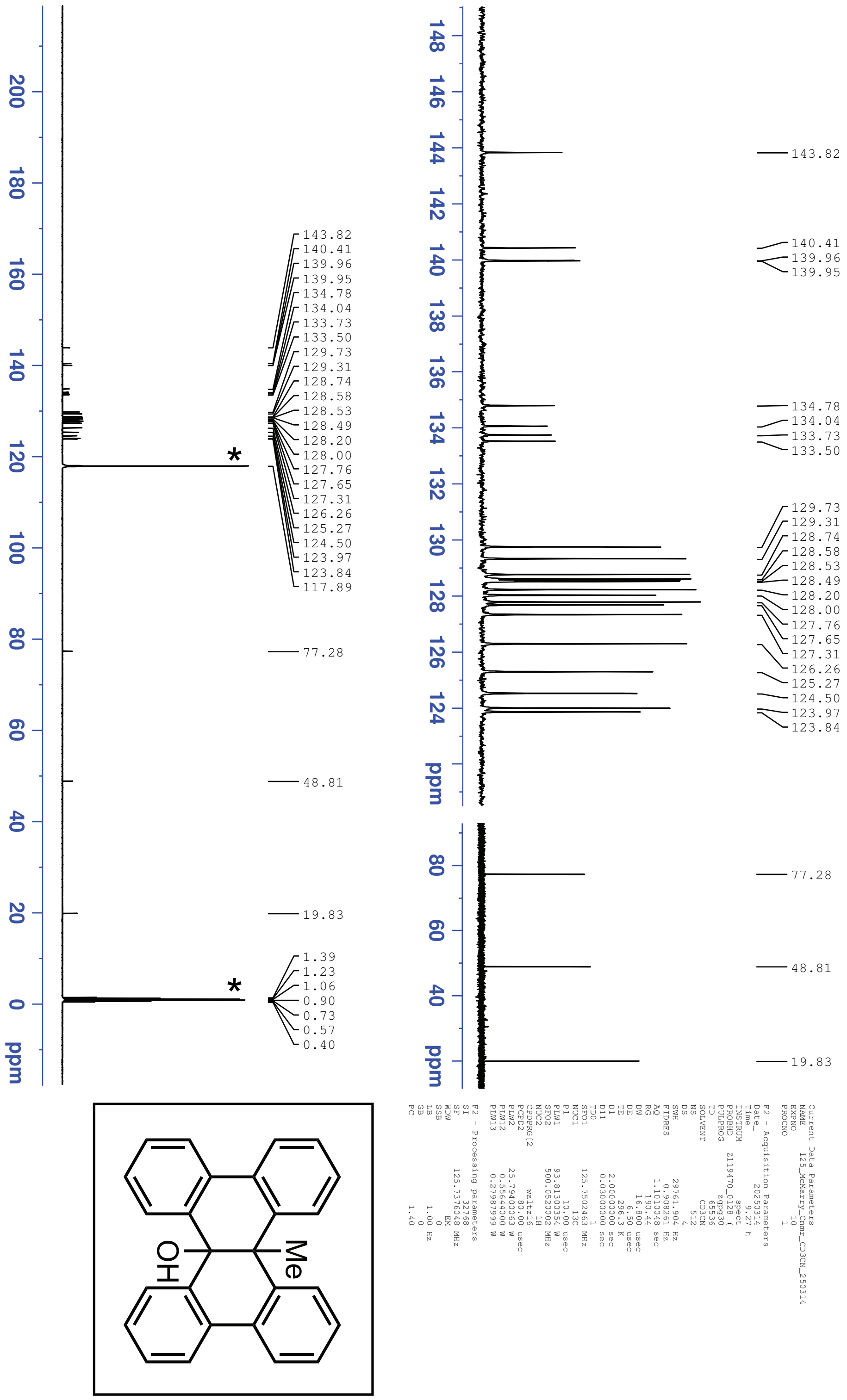

Figure S8. ^{13}C NMR spectrum of **6** in CD_3CN at 25 °C.

4. Mass spectra

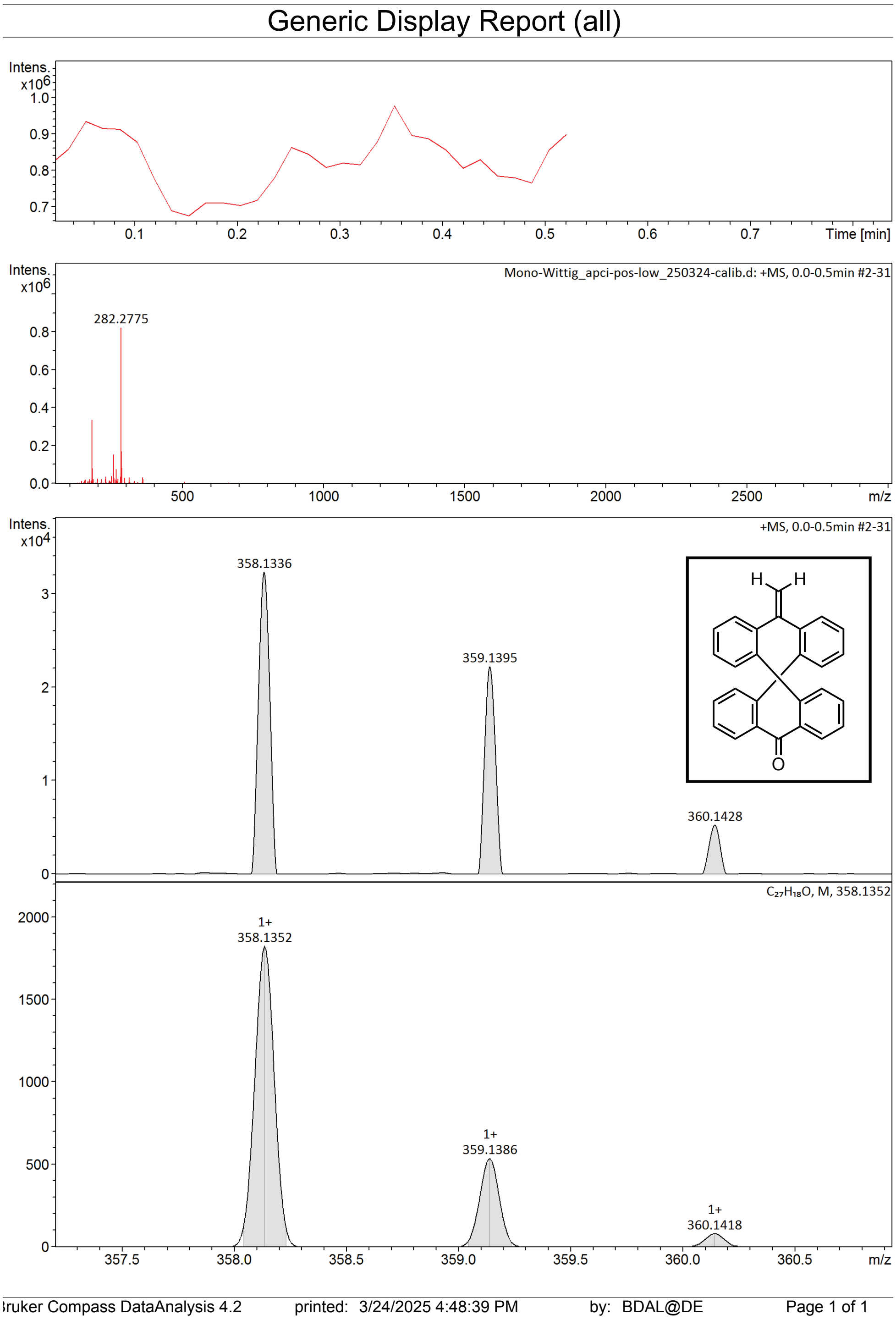

Figure S9. APCI-TOF mass spectrum of **3**.

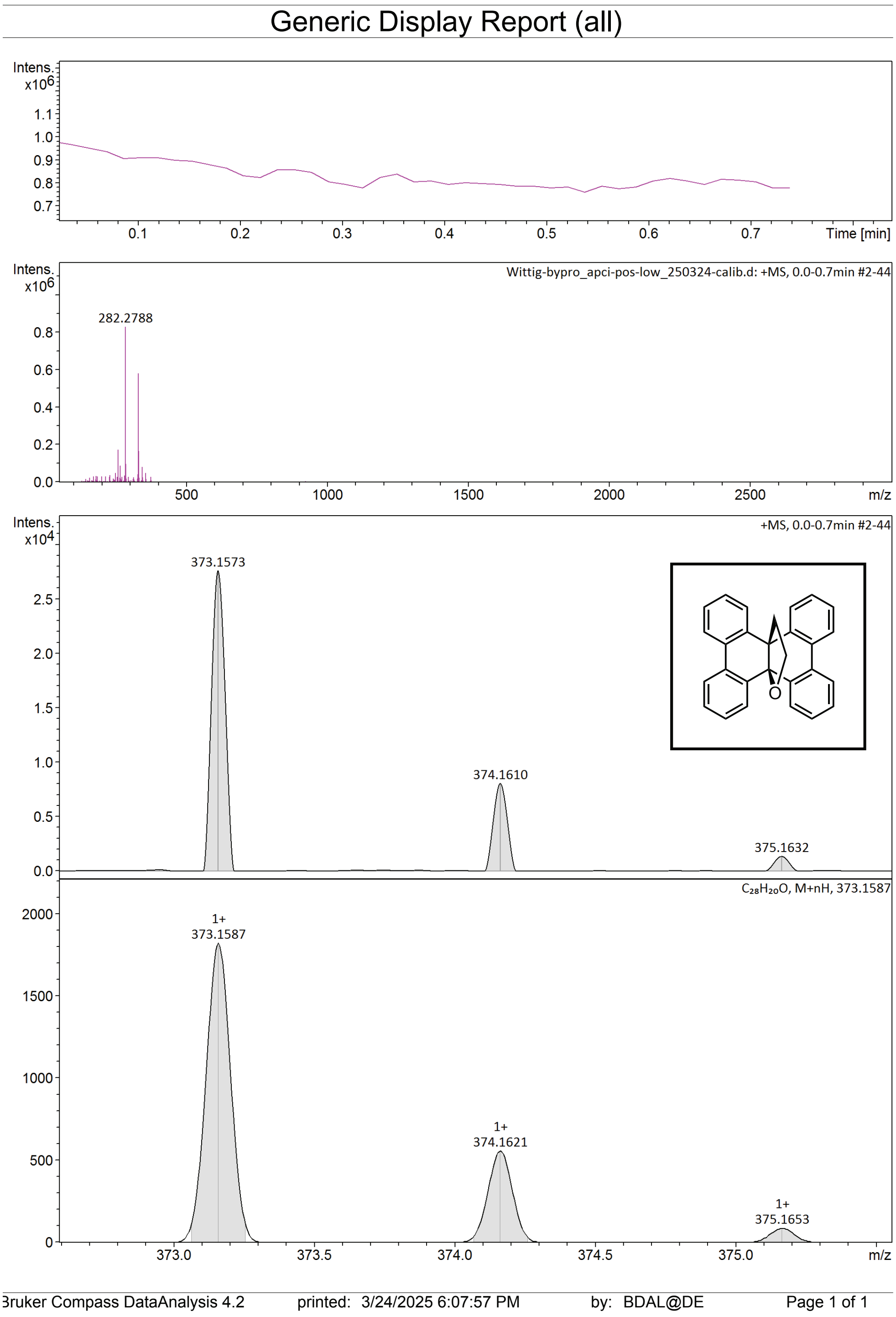

Figure S10. APCI-TOF mass spectrum of **4**.

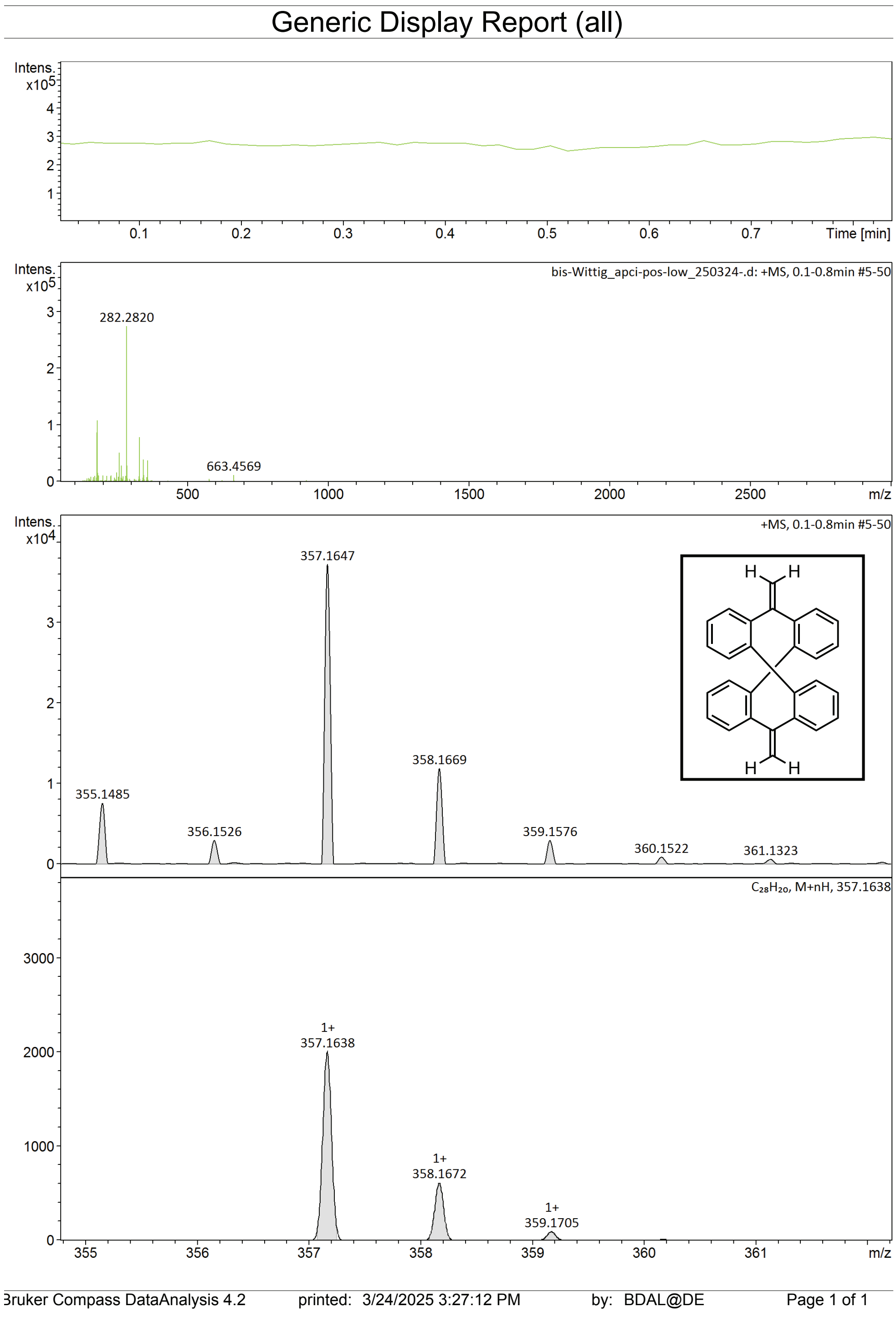

Figure S11. APCI-TOF mass spectrum of **5**.

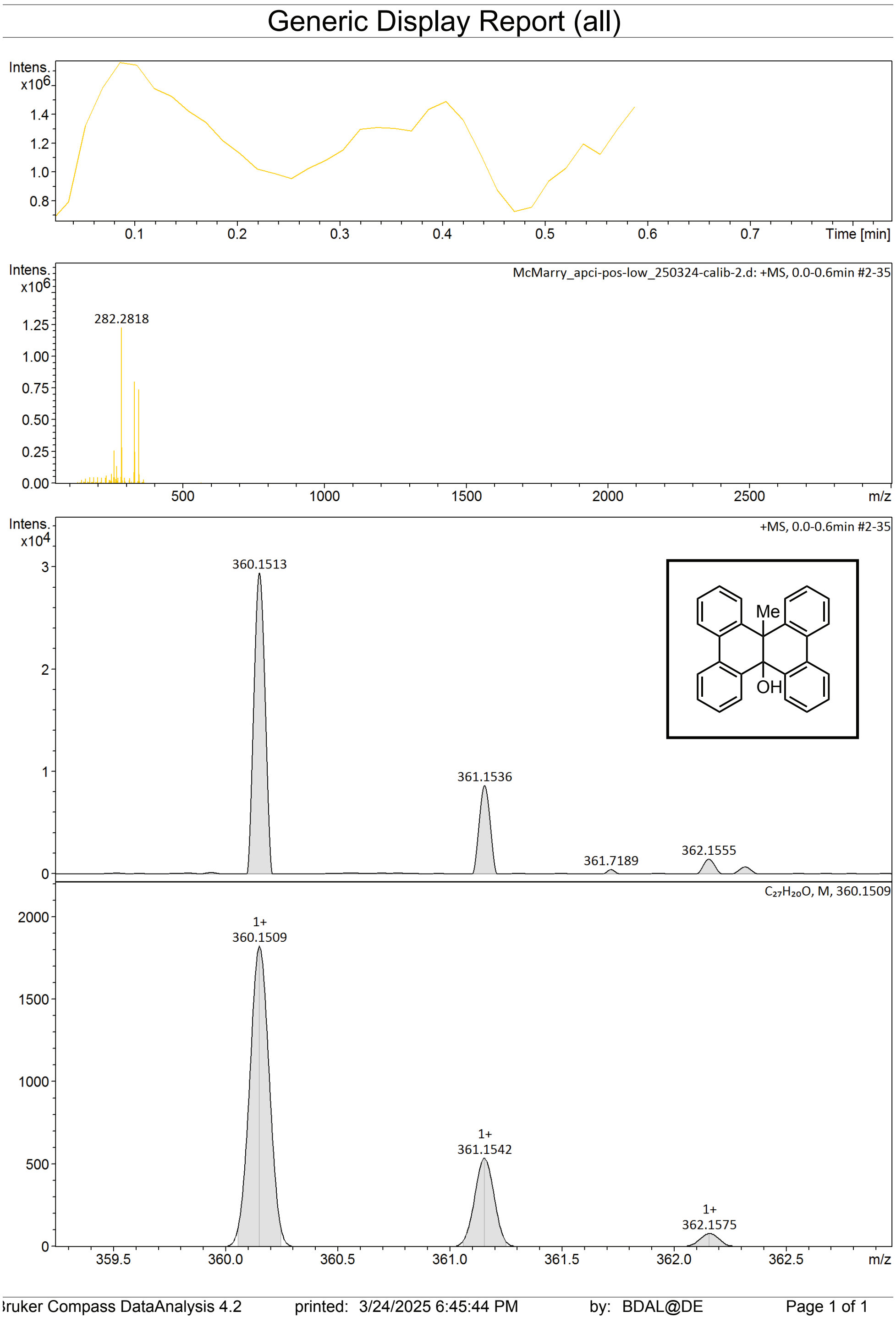

Figure S12. APCI-TOF mass spectrum of **6**.

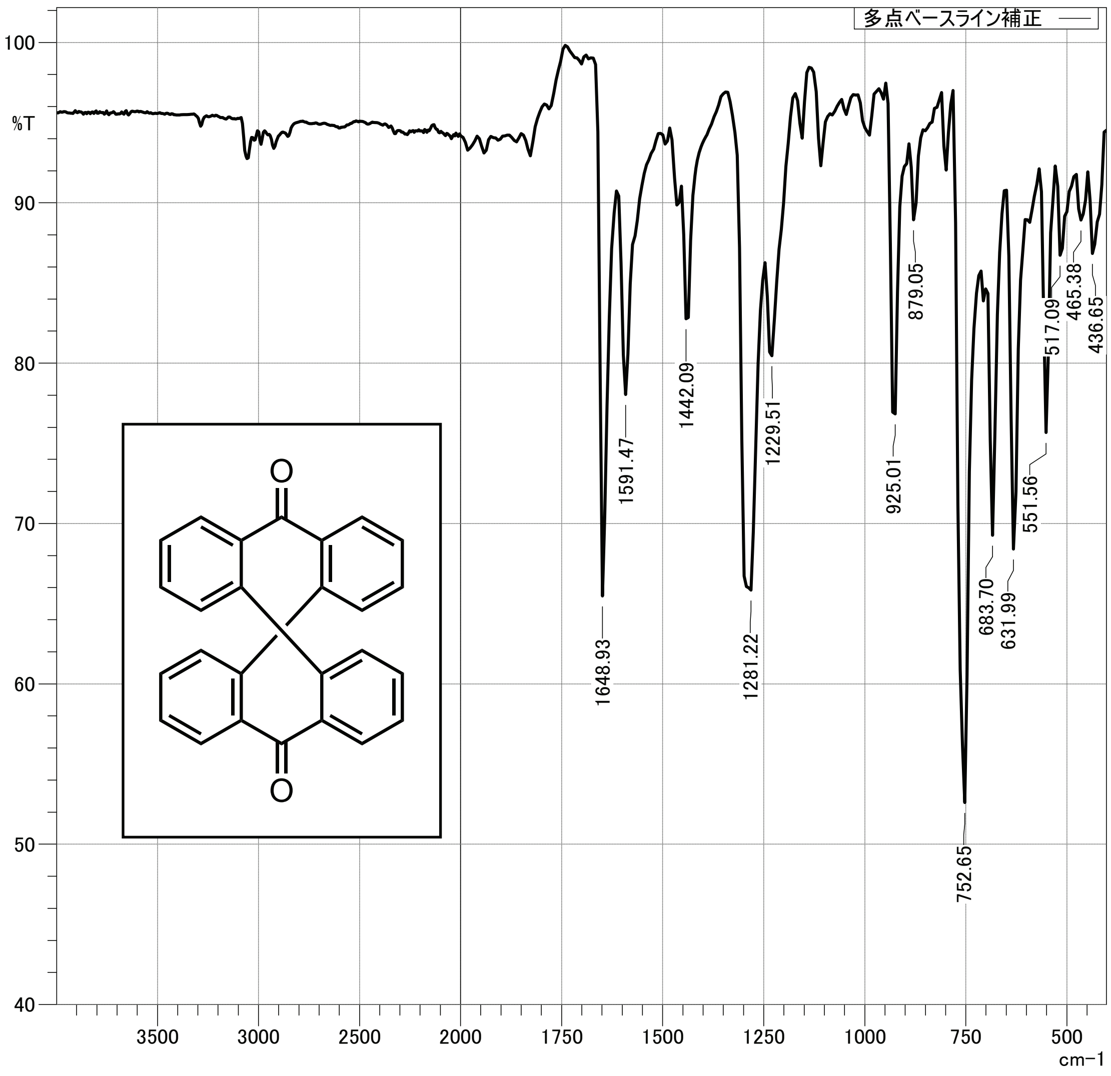

Figure S13. FT-IR spectrum of **1**.

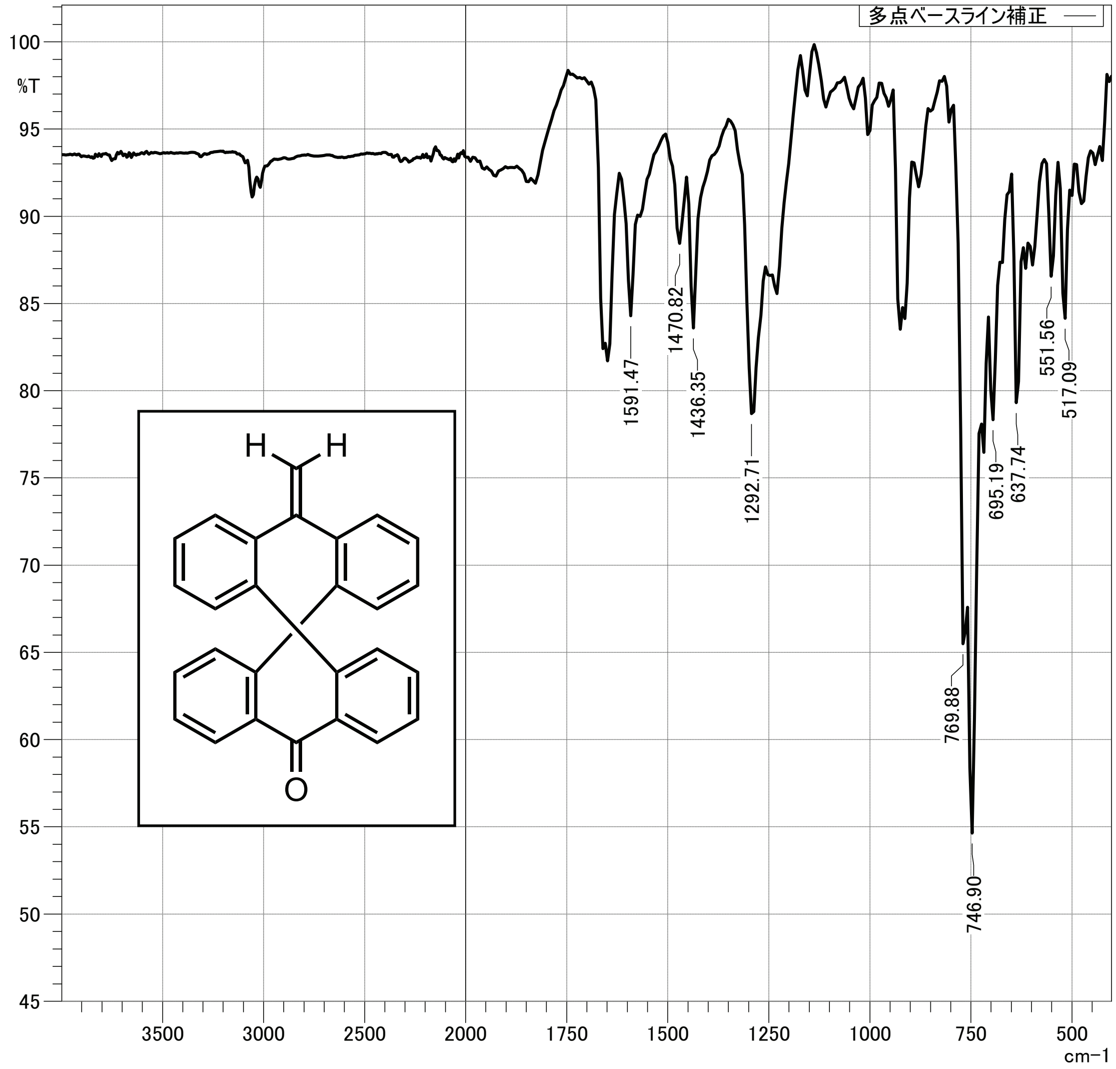

Figure S14. FT-IR spectrum of **3**.

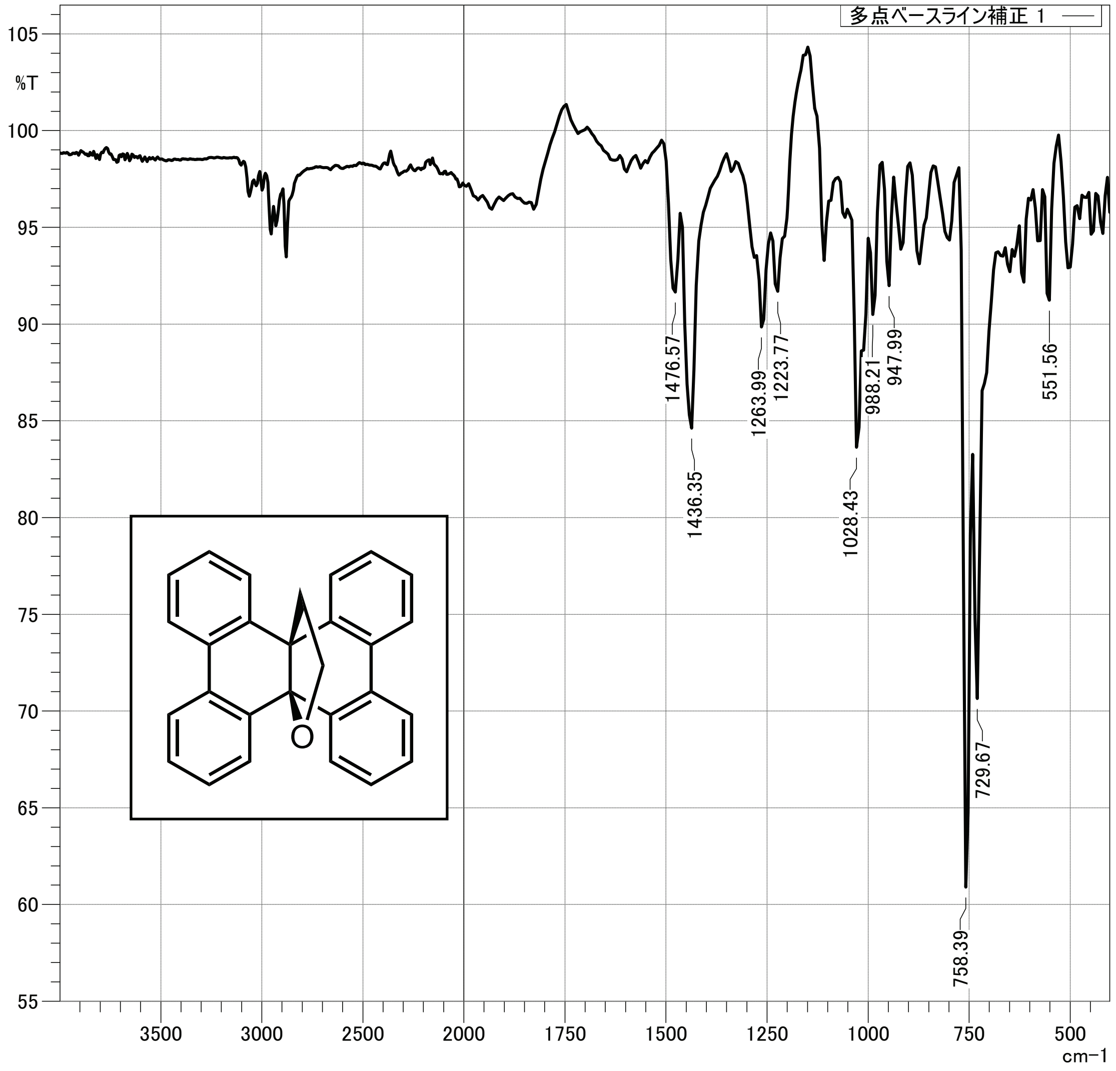

Figure S15. FT-IR spectrum of **4**.

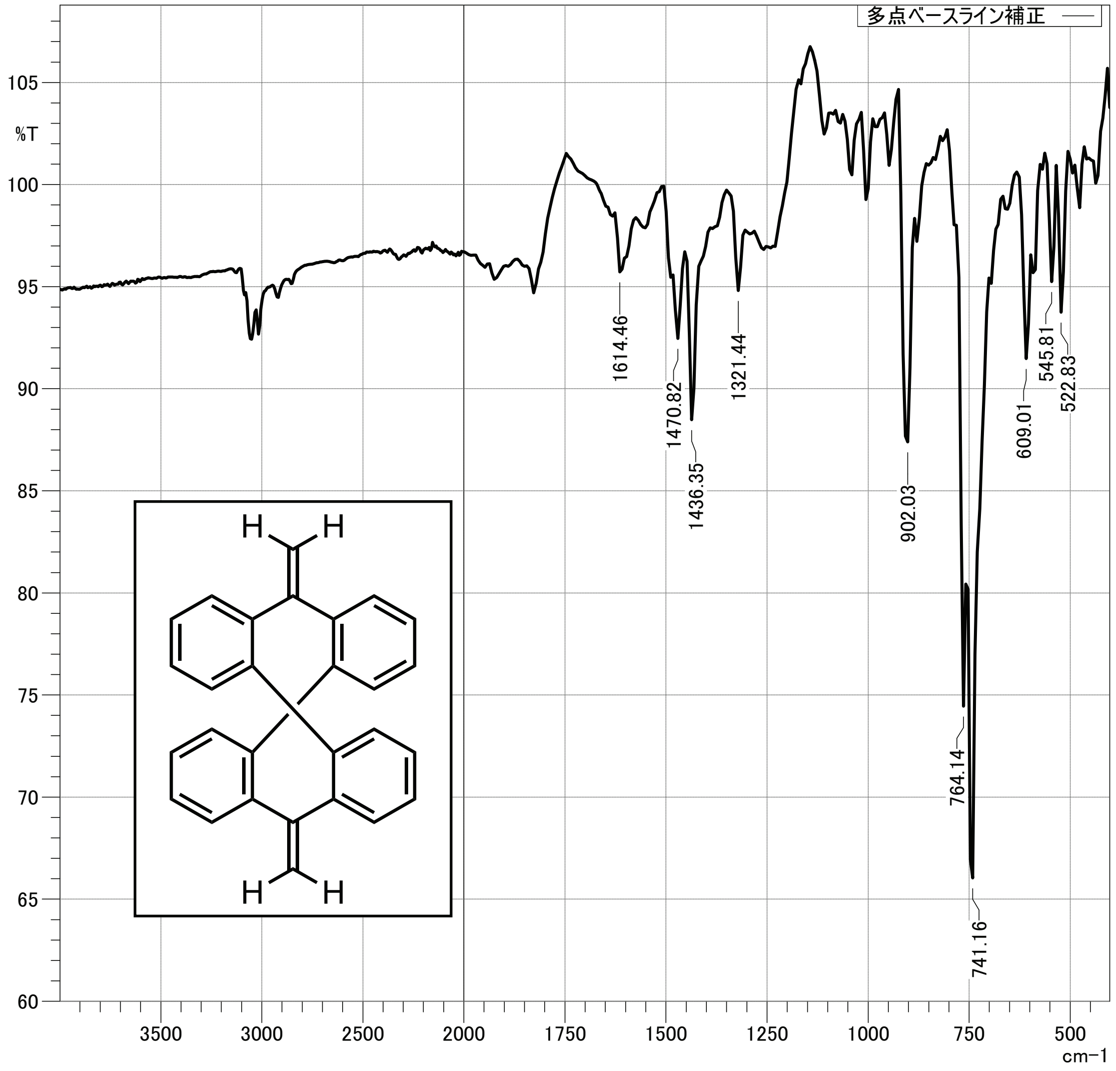

Figure S16. FT-IR spectrum of **5**.

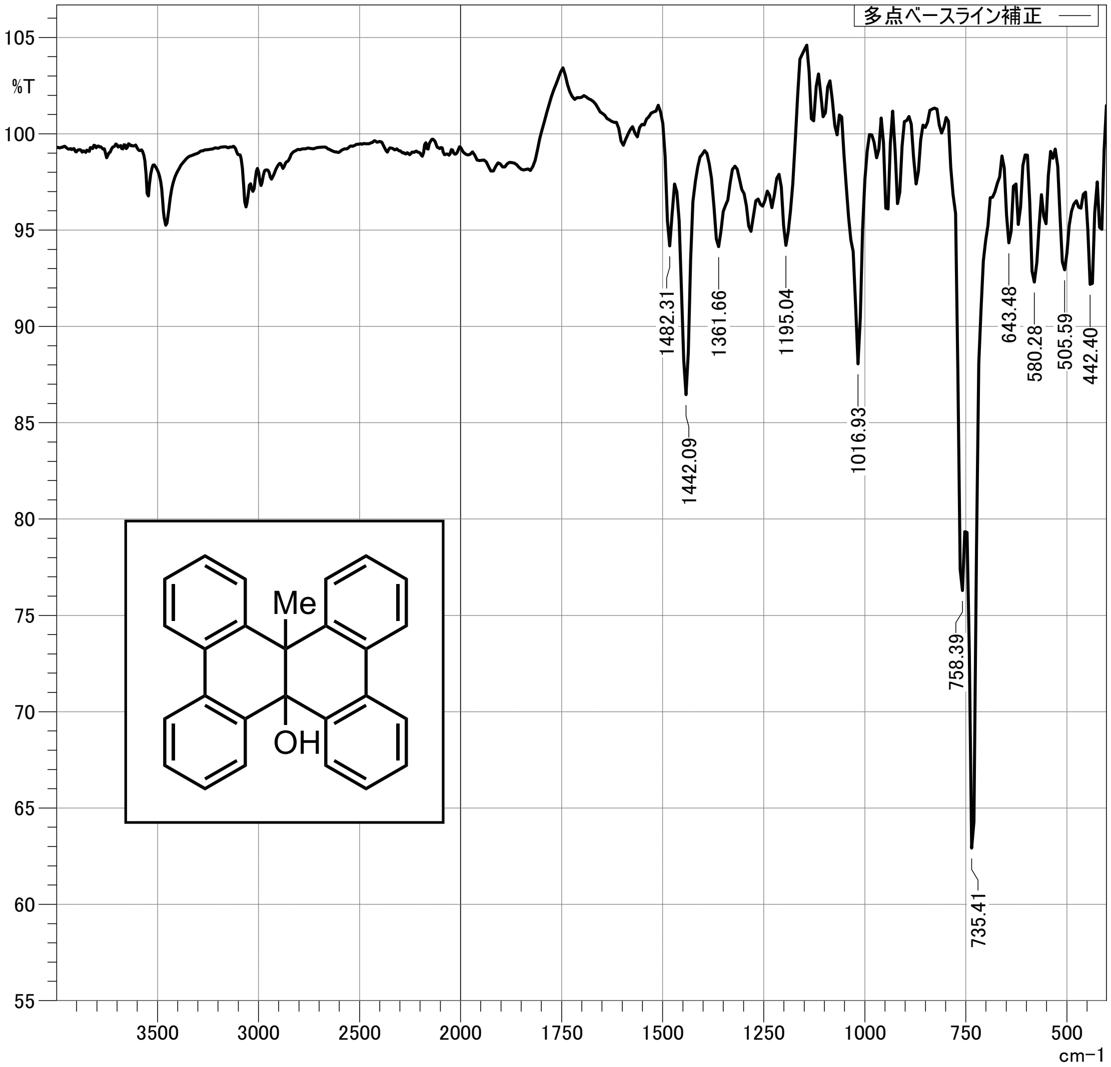

Figure S17. FT-IR spectrum of **6**.

5. Crystal data

Table S1. Crystallographic data of **3**, **4**, and **5**.

compound	**3**	**4**	**5**
Formula	$C_{27}H_{18}O$	$C_{28}H_{20}O$	$C_{28}H_{20}$
Formula weight	356.40	374.45	356.44
Crystal system	monoclinic	orthorhombic	monoclinic
Space group	$P2_1/c$ (No. 14)	*Pbcn* (No. 60)	*Cc* (No. 9)
Crystal color	colorless	colorless	colorless
Crystal description	block	block	plate
a [Å]	8.1475(3)	16.2309(3)	8.02857(14)
b [Å]	28.7163(10)	7.0737(1)	17.8783(3)
c [Å]	8.0068(3)	16.0738(3)	13.5369(9)
α [°]	—	—	—
β [°]	98.642(3)	—	98.7140(16)
γ [°]	—	—	—
V [Å^3]	1852.06(11)	1845.47(6)	1920.61(6)
Z	4	4	4
d_{calcd} [g cm^{-3}]	1.278	1.348	1.233
R_1 ($I > 2\sigma(I)$)	0.0410	0.0496	0.0290
wR_2 (all data)	0.1129	0.1307	0.0745
Goodness-of-fit	1.050	1.084	1.082
Temperature [K]	93(2)	93(2)	93(2)
CCDC No.	2432952	2432953	2432954

Table S2. Crystallographic data of **6**.

compound	**6**
Formula	$C_{27}H_{20}O$
Formula weight	360.43
Crystal system	trigonal
Space group	$P3_121$ (No. 152)
Crystal color	colorless
Crystal description	block
a [Å]	9.6005(2)
b [Å]	9.6005(2)
c [Å]	34.7275(6)
α [°]	—
β [°]	—
γ [°]	—
V [Å^3]	2771.99(13)
Z	6
d_{calcd} [g cm^{-3}]	1.295
R_1 ($I > 2\sigma(I)$)	0.0324
wR_2 (all data)	0.0793
Goodness-of-fit	1.051
Temperature [K]	93(2)
CCDC No.	2432955

6. DFT calculations

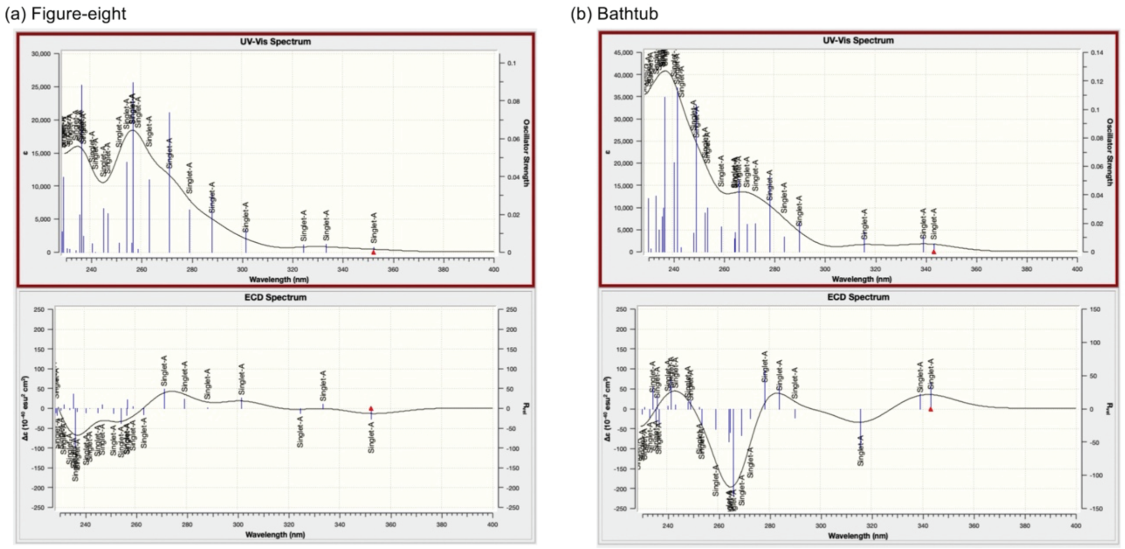

Figure S18. Simulated absorption (top) and CD (bottom) spectra of (a) (*M*,*M*)-figure-eight and (b) (*S*a,*S*a)-bathtub conformers of **3**. Half-width at half height = 0.13 eV.

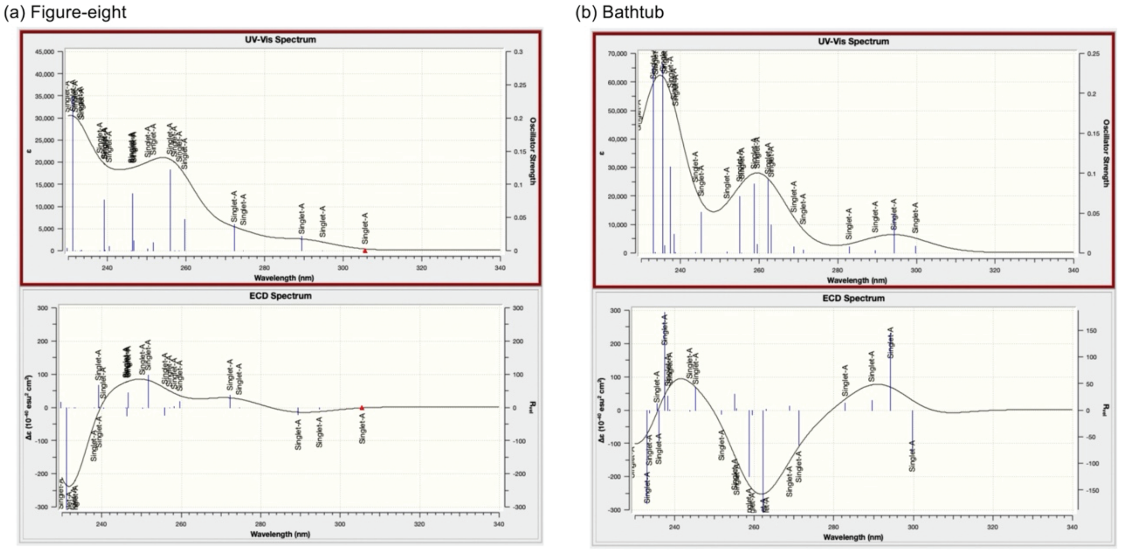

Figure S19. Simulated absorption (top) and CD (bottom) spectra of (a) (*M*,*M*)-figure-eight and (b) (*S*a,*S*a)-bathtub conformers of **5**. Half-width at half height = 0.13 eV

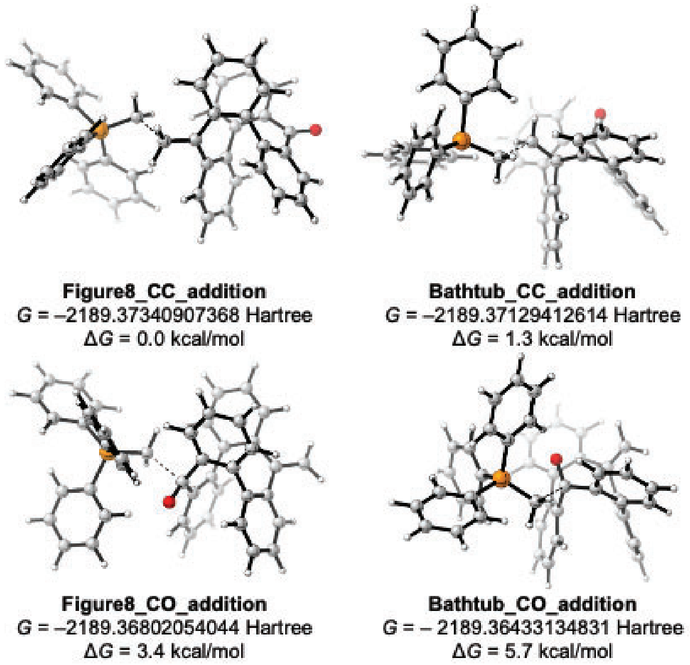

Figure S20. Transition state (TS) structures for the addition of methylenetriphenylphosphorane to the C=C or C=O bonds of **3**.

Table S3. Cartesian coordinate and geometry of (*M*,*M*)-**3** (figure-eight).

C	-1.004165	0.85962	1.488496
C	-0.74375	1.552963	0.286503
C	-1.764609	2.329837	-0.278119
H	-1.558237	2.876028	-1.194431
C	-3.018765	2.42477	0.322587
H	-3.794198	3.031047	-0.138335
C	-3.267217	1.755437	1.522149
H	-4.237873	1.83273	2.004238
C	-2.260547	0.990077	2.101111
H	-2.428614	0.47091	3.039077
C	1.00389	-0.855588	1.490848
C	0.743661	-1.552133	0.290664
C	1.764638	-2.330436	-0.271776
H	1.558405	-2.879079	-1.186653
C	3.018735	-2.423682	0.329318
H	3.794261	-3.031129	-0.129905
C	3.267009	-1.751141	1.527121
H	4.237619	-1.827081	2.009519
C	2.260219	-0.984323	2.103938
H	2.428144	-0.462655	3.040541
C	-0.000207	0.002932	2.215646
O	-0.000208	0.004612	3.442025
C	0.954315	0.890159	-1.51665
C	0.616283	1.595838	-0.339318
C	1.57034	2.429844	0.261568
H	1.291563	2.974649	1.15958
C	2.848466	2.579212	-0.271946
H	3.568463	3.23169	0.214376
C	3.192197	1.876329	-1.425706
H	4.189482	1.964155	-1.848394
C	2.256299	1.040543	-2.027882
H	2.541324	0.460695	-2.900225
C	-0.954199	-0.894263	-1.514476
C	-0.61629	-1.596775	-0.335213
C	-1.570366	-2.429239	0.26778
H	-1.291663	-2.971636	1.167272
C	-2.848423	-2.580091	-0.265475
H	-3.568447	-3.231301	0.222504
C	-3.192074	-1.88025	-1.421106
H	-4.189329	-1.969197	-1.84363
C	-2.256154	-1.04602	-2.025398
H	-2.541172	-0.468429	-2.899234
C	0.000105	-0.002972	-2.244124
C	0.000622	-0.005037	-3.592059
H	-0.622708	-0.687614	-4.160891
H	0.624571	0.675656	-4.162474

Table S4. Cartesian coordinate and geometry of (*S*a,*S*a)-**3** (bathtub).

O	-0.046238	-1.871463	-1.890856
C	-1.484403	0.69167	-1.078557
C	0.497563	-0.886768	-1.414203
C	1.881114	-0.985569	-0.831395
C	-0.159936	0.463883	-1.507489
C	0.552371	1.477809	-2.160318
H	1.574456	1.284304	-2.474915
C	0.158925	0.382851	1.54907
C	2.317741	-0.264373	0.307025
C	-2.311186	-0.310009	-0.342227
C	-0.487896	-0.953807	1.360041
C	1.464573	0.684403	1.088754
C	2.74957	-1.886122	-1.464033
H	2.371716	-2.443012	-2.315767
C	2.004569	1.947335	1.388543
H	2.995799	2.19129	1.016805
C	-3.62639	-0.507372	-0.796699
H	-3.94966	0.022248	-1.688113
C	-2.049823	1.950756	-1.339997
H	-3.055026	2.155297	-0.982881
C	-1.870276	-1.036228	0.795856
C	1.290785	2.899222	2.111584
H	1.736179	3.868568	2.318425
C	-0.552751	1.359606	2.261754
H	-1.556319	1.122585	2.604226
C	3.63819	-0.46795	0.743001
H	3.978856	0.053342	1.633147
C	-0.035817	2.711419	-2.429998
H	0.525709	3.481006	-2.952283
C	-1.345748	2.946412	-2.014974
H	-1.81676	3.907963	-2.200882
C	0.142383	-2.070564	1.76269
C	4.058359	-2.059523	-1.023936
H	4.721001	-2.750904	-1.536973
C	0.000495	2.603996	2.550499
H	-0.57218	3.335955	3.113623
C	-2.782632	-1.896906	1.432841
H	-2.45857	-2.41635	2.329433
C	-4.505939	-1.379913	-0.162559
H	-5.513077	-1.510962	-0.548804
C	4.503497	-1.341587	0.086116
H	5.51861	-1.470065	0.452617
C	-4.081689	-2.076607	0.967297
H	-4.756529	-2.750014	1.488771
H	1.124581	-2.031826	2.22281
H	-0.292773	-3.054117	1.615497

Table S5. Cartesian coordinate and geometry of (*M*,*M*)-**5** (figure-eight).

C	-0.993983	-0.85924	-1.463715
C	-0.682674	-1.608529	-0.305536
C	-1.664182	-2.423871	0.273537
H	-1.403822	-3.000468	1.157114
C	-2.947826	-2.510692	-0.261041
H	-3.691223	-3.150622	0.206824
C	-3.267252	-1.761268	-1.392425
H	-4.268168	-1.799418	-1.814246
C	-2.301639	-0.945254	-1.975191
H	-2.565474	-0.33384	-2.832568
C	0.993808	0.858865	-1.464056
C	0.682689	1.608383	-0.305969
C	1.664319	2.423746	0.27287
H	1.404089	3.000522	1.156369
C	2.947922	2.510359	-0.261834
H	3.69142	3.150298	0.205858
C	3.267188	1.760675	-1.393089
H	4.268085	1.798617	-1.814971
C	2.301455	0.944636	-1.97562
H	2.565213	0.332968	-2.832833
C	-0.000167	-0.000255	-2.184322
C	0.99397	-0.858874	1.463947
C	0.682665	-1.608395	0.305932
C	1.664214	-2.423812	-0.273015
H	1.403822	-3.000606	-1.156451
C	2.947879	-2.510434	0.261518
H	3.691321	-3.150393	-0.206236
C	3.267307	-1.760768	1.39274
H	4.268252	-1.798752	1.814507
C	2.30166	-0.944712	1.975388
H	2.565494	-0.333108	2.83262
C	-0.993827	0.859232	1.463844
C	-0.682588	1.608482	0.305638
C	-1.664131	2.423862	-0.27337
H	-1.403786	3.000457	-1.15695
C	-2.947734	2.510694	0.26128
H	-3.691174	3.150616	-0.206527
C	-3.267101	1.761285	1.392689
H	-4.267997	1.799438	1.814557
C	-2.301455	0.945278	1.975407
H	-2.565239	0.333885	2.832805
C	0.000087	0.00024	2.18436
C	0.000138	0.000386	3.532373
H	-0.651577	0.655651	4.101696
H	0.651896	-0.654756	4.10179
C	-0.000631	-0.000106	-3.532336
H	-0.652624	-0.65522	-4.101524
H	0.650828	0.655249	-4.101853

Table S6. Cartesian coordinate and geometry of (*S*a,*S*a)-**5** (bathtub).

C	3.599402	-0.480852	0.823159
H	3.894793	0.044221	1.727256
C	4.510417	-1.327393	0.195777
H	5.511381	-1.44447	0.602402
C	1.893184	-1.009961	-0.82063
C	1.432774	0.679628	1.086697
C	0.526703	-0.928821	-1.41796
C	0.127687	0.406556	1.557961
C	-2.29105	-0.295931	-0.342183
C	-1.893174	-1.01028	0.820213
C	2.291127	-0.296303	0.342172
C	-1.432673	0.680356	-1.086197
C	0.080541	-2.030618	1.890188
C	-0.127643	0.407374	-1.557717
C	1.97817	1.95191	1.33854
H	2.970427	2.176114	0.957442
C	-0.526769	-0.929512	1.417653
C	-1.270983	2.934949	-2.02512
H	-1.722164	3.909116	-2.193856
C	4.125519	-2.012627	-0.954497
H	4.824148	-2.665171	-1.471279
C	-2.834908	-1.845086	1.447902
H	-2.542086	-2.35238	2.362129
C	-4.125419	-2.013193	0.953312
H	-4.82401	-2.666195	1.469566
C	0.579328	1.413106	-2.23518
H	1.58394	1.193471	-2.586576
C	-0.081109	-2.029709	-1.890335
C	-1.97801	1.952739	-1.337538
H	-2.970214	2.176904	-0.956269
C	-0.57938	1.412204	2.2355
H	-1.584094	1.192554	2.586578
C	-3.599313	-0.480223	-0.82334
H	-3.894677	0.045404	-1.727119
C	-4.510305	-1.327219	-0.196536
H	-5.511251	-1.444102	-0.603261
C	2.834967	-1.844267	-1.448932
H	2.542189	-2.350932	-2.363519
C	1.27111	2.933939	2.026317
H	1.722298	3.908026	2.195502
C	0.020218	2.663986	-2.477585
H	0.588207	3.419567	-3.013744
C	-0.020228	2.662947	2.478401
H	-0.588262	3.418475	3.014591
H	-1.053448	-1.976621	-2.369346
H	0.361421	-3.015851	-1.785416
H	-0.362408	-3.016546	1.785116
H	1.052707	-1.977767	2.369536

Table S7. Cartesian coordinate and geometry of TS1 of **5**.

C	1.258	1.307	-0.813
C	2.356	0.436	-0.607
C	3.348	0.346	-1.588
H	4.194	-0.318	-1.426
C	3.232	1.047	-2.79
H	4.007	0.956	-3.548
C	2.107	1.836	-3.028
H	1.998	2.366	-3.971
C	1.131	1.97	-2.039
H	0.277	2.623	-2.201
C	-1.133	1.752	0.333
C	-2.1	0.7	0.299
C	-3.358	1.019	0.878
H	-4.063	0.212	1.035
C	-3.747	2.296	1.264
H	-4.735	2.452	1.688
C	-2.863	3.352	1.083
H	-3.142	4.375	1.321
C	-1.575	3.056	0.656
H	-0.842	3.854	0.601
C	0.367	1.658	0.332
C	1.258	-1.307	0.813
C	2.357	-0.436	0.607
C	3.348	-0.346	1.588
H	4.195	0.318	1.426
C	3.233	-1.047	2.79
H	4.007	-0.955	3.548
C	2.107	-1.836	3.028
H	1.999	-2.365	3.971
C	1.131	-1.97	2.039
H	0.277	-2.623	2.201
C	-1.133	-1.753	-0.333
C	-2.1	-0.7	-0.299
C	-3.358	-1.02	-0.878
H	-4.063	-0.213	-1.035
C	-3.747	-2.296	-1.264
H	-4.735	-2.453	-1.688
C	-2.863	-3.352	-1.084
H	-3.142	-4.375	-1.321
C	-1.575	-3.056	-0.657
H	-0.841	-3.854	-0.601
C	0.367	-1.658	-0.332
C	0.957	2.087	1.464
H	2.036	2.14	1.559
H	0.376	2.376	2.334
C	0.957	-2.087	-1.464
H	0.376	-2.376	-2.334
H	2.037	-2.14	-1.559

Table S8. Cartesian coordinate and geometry of TS2 of **5**.

C	0.37	0.641	1.597
C	1.406	0.977	0.697
C	1.861	2.301	0.645
H	2.666	2.553	-0.039
C	1.294	3.289	1.45
H	1.659	4.311	1.392
C	0.263	2.958	2.329
H	-0.182	3.719	2.965
C	-0.19	1.641	2.399
H	-0.982	1.375	3.095
C	-1.249	-1.369	1.115
C	-1.89	-0.797	-0.014
C	-2.977	-1.472	-0.598
H	-3.45	-1.022	-1.467
C	-3.459	-2.677	-0.1
H	-4.3	-3.17	-0.579
C	-2.863	-3.22	1.038
H	-3.24	-4.142	1.474
C	-1.79	-2.568	1.628
H	-1.369	-2.986	2.536
C	-0.044	-0.794	1.785
C	1.565	-0.757	-1.227
C	2.095	-0.1	-0.086
C	3.33	-0.523	0.435
H	3.731	0.01	1.293
C	4.021	-1.612	-0.085
H	4.969	-1.918	0.349
C	3.453	-2.326	-1.14
H	3.943	-3.212	-1.536
C	2.252	-1.9	-1.691
H	1.824	-2.477	-2.503
C	-0.554	0.793	-1.533
C	-1.593	0.548	-0.613
C	-2.506	1.577	-0.333
H	-3.316	1.375	0.362
C	-2.398	2.835	-0.923
H	-3.117	3.612	-0.68
C	-1.367	3.08	-1.828
H	-1.268	4.052	-2.304
C	-0.464	2.063	-2.128
H	0.334	2.242	-2.842
C	0.399	-0.261	-2.025
C	0.748	-1.537	2.579
H	1.611	-1.094	3.065
H	0.598	-2.598	2.748
C	0.22	-0.637	-3.307
H	-0.62	-0.256	-3.878
H	0.892	-1.305	-3.834

Table S9. Cartesian coordinate and geometry of conformer **C** of **5**.

C	1.298	1.6	-0.282
C	2.34	0.644	-0.376
C	3.438	0.898	-1.213
H	4.222	0.148	-1.279
C	3.526	2.058	-1.979
H	4.385	2.225	-2.623
C	2.489	2.985	-1.918
H	2.523	3.893	-2.514
C	1.397	2.748	-1.086
H	0.604	3.485	-1.064
C	-1.244	1.718	0.117
C	-2.209	0.729	-0.194
C	-3.397	1.155	-0.829
H	-4.119	0.399	-1.126
C	-3.682	2.492	-1.079
H	-4.61	2.766	-1.574
C	-2.784	3.468	-0.655
H	-3.005	4.524	-0.781
C	-1.586	3.07	-0.07
H	-0.895	3.829	0.284
C	0.128	1.421	0.652
C	1.299	-1.6	0.282
C	2.34	-0.644	0.376
C	3.438	-0.898	1.213
H	4.222	-0.147	1.279
C	3.526	-2.058	1.979
H	4.386	-2.224	2.623
C	2.49	-2.984	1.918
H	2.524	-3.893	2.514
C	1.397	-2.748	1.086
H	0.605	-3.485	1.064
C	-1.243	-1.718	-0.117
C	-2.209	-0.729	0.194
C	-3.397	-1.155	0.829
H	-4.118	-0.4	1.126
C	-3.682	-2.493	1.079
H	-4.61	-2.767	1.574
C	-2.783	-3.468	0.655
H	-3.004	-4.524	0.781
C	-1.585	-3.07	0.07
H	-0.894	-3.829	-0.284
C	0.128	-1.421	-0.652
C	0.29	1.083	1.938
H	1.269	0.898	2.368
H	-0.565	0.976	2.6
C	0.29	-1.083	-1.938
H	-0.565	-0.976	-2.6
H	1.269	-0.898	-2.368

Table S10. Cartesian coordinate and geometry of TS **Figure8_CO**.

C	1.315854622	0.7410455	1.091726103
C	2.673990064	0.748704531	1.501293151
C	3.197515418	1.873564293	2.160539371
H	4.240668607	1.860180374	2.46158928
C	2.403751001	2.989158283	2.434947206
H	2.826363989	3.848279751	2.946889555
C	1.062089875	2.97938165	2.048271916
H	0.423145414	3.831853121	2.258518657
C	0.532579561	1.864346303	1.391604454
H	-0.511161752	1.868182278	1.115764635
C	1.401754768	-1.210213967	-0.690703281
C	2.156985433	-0.551285748	-1.696227047
C	2.698749702	-1.309821893	-2.752783516
H	3.274612222	-0.792948439	-3.514589649
C	2.52070676	-2.691531011	-2.835858868
H	2.949670226	-3.247749719	-3.663829287
C	1.795967436	-3.344513851	-1.834576342
H	1.65997285	-4.421447445	-1.867763521
C	1.25658015	-2.607618723	-0.781629318
H	0.71838389	-3.09981951	0.017881157
C	0.731762375	-0.561313769	0.527657808
O	0.280998031	-1.365992135	1.440264806
C	4.555543324	-0.432026929	0.252741333
C	3.578301667	-0.423766717	1.278027469
C	3.467029124	-1.529662284	2.138301676
H	2.692463454	-1.517560819	2.89634688
C	4.308474959	-2.636312548	2.008656361
H	4.203083517	-3.480436772	2.683002392
C	5.270438034	-2.654881649	0.993944325
H	5.918009369	-3.516979143	0.866103076
C	5.381178884	-1.568675123	0.125443923
H	6.095604015	-1.605308339	-0.689793711
C	3.628761111	1.505297617	-1.269705432
C	2.423024996	0.927046238	-1.747386131
C	1.491308311	1.747445283	-2.40853809
H	0.600347542	1.285947365	-2.820862073
C	1.691730706	3.121617333	-2.558275136
H	0.951317151	3.728225097	-3.070876608
C	2.850421254	3.703447093	-2.038107469
H	3.015909952	4.77313796	-2.121167135
C	3.79925979	2.899555282	-1.406350356
H	4.690307265	3.354116165	-0.987798766
C	4.764851888	0.714715051	-0.687686752
C	6.028357186	1.049924826	-1.038272886
H	6.232042693	1.821423185	-1.772635548
H	6.889424176	0.557118331	-0.600409069
C	-0.997407193	0.14397972	-0.693670153
H	-0.86647084	-0.516343476	-1.551777042
H	-0.760699731	1.183737994	-0.917918676
P	-2.714017988	-0.03267901	-0.103638019
C	-3.156836806	-1.857989846	-0.081987071
C	-2.952843857	0.665763345	1.627697593
C	-4.01595867	0.811450848	-1.187734781

C	-2.28054233	-2.740346131	0.571328622
C	-4.314862234	-2.344902187	-0.707680799
C	-2.155971977	0.149993496	2.665686366
C	-3.880667176	1.685002766	1.892937561
C	-3.581956682	1.453582798	-2.354929227
C	-5.386848772	0.798521153	-0.871562777
C	-2.587534345	-4.105929823	0.613009376
H	-1.353970115	-2.373625702	1.0145615
C	-4.607299799	-3.713116518	-0.662545626
H	-4.981799172	-1.678122344	-1.239159423
C	-2.324984629	0.635100417	3.967359143
H	-1.367343278	-0.56952889	2.45474327
C	-4.035964075	2.165768811	3.198936228
H	-4.473734215	2.116170239	1.096472127
C	-4.505075321	2.081271051	-3.200605037
H	-2.524401592	1.456828337	-2.600193883
C	-6.307375259	1.425198969	-1.719151624
H	-5.739173259	0.305374409	0.028642912
C	-3.748468642	-4.59424488	0.003102969
H	-1.909386223	-4.78614863	1.118256978
H	-5.501923836	-4.085691766	-1.151339885
C	-3.267033677	1.6340008	4.2391001
H	-1.703575941	0.239598127	4.763919519
H	-4.753020241	2.956219309	3.396781019
C	-5.86731743	2.067824933	-2.883692404
H	-4.159425813	2.576281461	-4.102255615
H	-7.363694746	1.411546454	-1.470333761
H	-3.978151484	-5.654653857	0.037578802
H	-3.389891351	2.006482604	5.251257893
H	-6.583405458	2.553439803	-3.5386826

Table S11. Cartesian coordinate and geometry of TS **Figure8_CC**.

C	3.951930058	1.586705677	-1.033330804
C	2.649007986	1.07599907	-1.2492622
C	1.620094085	1.981330518	-1.565449917
H	0.627437398	1.588138624	-1.760576353
C	1.858859603	3.353494459	-1.666853243
H	1.043867213	4.02863247	-1.910406165
C	3.153815686	3.850564051	-1.476063174
H	3.352946472	4.913749944	-1.565015602
C	4.188574275	2.967914382	-1.175023353
H	5.201845567	3.327528959	-1.036481383
C	5.038571853	-0.490775809	0.132357684
C	4.146171754	-0.620576004	1.222695323
C	4.113699205	-1.842234416	1.919762301
H	3.431072987	-1.941566964	2.757242891
C	4.951406805	-2.902419538	1.568413298
H	4.906558685	-3.833950746	2.124804714
C	5.863369707	-2.753789489	0.514959674
H	6.529809269	-3.56745097	0.24676238
C	5.907516644	-1.552520917	-0.188318078
H	6.605359466	-1.41204679	-1.006226746
C	5.14418793	0.738486515	-0.710071594
O	6.263365036	1.069467723	-1.173272909
C	1.584776271	-1.056126582	-0.282401015
C	2.341931203	-0.391578653	-1.293285318
C	2.759026481	-1.094372683	-2.438694495
H	3.332755447	-0.561590373	-3.19179394
C	2.43625993	-2.438068282	-2.637375387
H	2.772928958	-2.957139047	-3.529206612
C	1.685138693	-3.103332376	-1.658896425
H	1.442988877	-4.156166912	-1.775587207
C	1.281796225	-2.425034385	-0.510327233
H	0.756107042	-2.97340338	0.2649694
C	1.92136655	0.587446429	1.6913734
C	3.347522466	0.527358289	1.761886394
C	4.061640688	1.509495462	2.47032713
H	5.144847291	1.433878666	2.506779941
C	3.417250274	2.543833837	3.152029776
H	3.992621878	3.285577008	3.696478703
C	2.019115648	2.594027052	3.126494208
H	1.49045162	3.386761437	3.649054204
C	1.297767656	1.643691	2.407324327
H	0.219374025	1.737009467	2.363490855
C	1.108718009	-0.398033957	0.958355142
C	-0.202018383	-0.685576676	1.405069408
H	-0.432591446	-0.486548198	2.448777107
H	-0.680539461	-1.585955618	1.036160675
P	-3.269562468	0.126766511	0.114731505
C	-1.598963329	0.63195406	0.673440504
H	-1.06847743	1.088581499	-0.164439936
H	-1.699822215	1.346385537	1.495089569
C	-3.942139567	-1.112976258	1.34753441
C	-3.510829238	-1.068890004	2.682290544
C	-4.875099887	-2.080995574	0.945489127

C	-4.018305699	-1.984997325	3.610813403
H	-2.777683651	-0.334723917	2.995316259
C	-5.376016082	-2.997654162	1.876501665
H	-5.199864918	-2.131970032	-0.088327491
C	-4.950204952	-2.949332616	3.209298002
H	-3.681112452	-1.947824542	4.641347419
H	-6.091615456	-3.749267078	1.559702536
H	-5.338031463	-3.662373047	3.929403187
C	-4.653325119	1.409813169	-0.069744982
C	-5.695218757	1.502491379	0.866231745
C	-4.540849988	2.37709923	-1.084982984
C	-6.626020292	2.545388045	0.77617605
H	-5.786678941	0.765914434	1.656829368
C	-5.476337128	3.410824581	-1.174660712
H	-3.728556687	2.320943178	-1.803649592
C	-6.520682281	3.497951646	-0.243030213
H	-7.432214874	2.609101425	1.500126565
H	-5.389783746	4.148162511	-1.966275932
H	-7.242849908	4.304965999	-0.310631114
C	-3.123968543	-0.68534081	-1.55574176
C	-1.893443322	-1.230926289	-1.953588008
C	-4.240546305	-0.776365538	-2.405253133
C	-1.780093167	-1.858769887	-3.199351354
H	-1.02551437	-1.171114055	-1.309110107
C	-4.120194482	-1.409677797	-3.646883296
H	-5.189882643	-0.341267524	-2.111862454
C	-2.891199054	-1.950419881	-4.04540462
H	-0.821400844	-2.270566264	-3.495281223
H	-4.983042022	-1.474634415	-4.301871396
H	-2.800805754	-2.438023765	-5.010657326

Table S12. Cartesian coordinate and geometry of TS **Bathtub_CO**.

O	1.306562073	-0.576297507	-2.048091715
C	2.401712697	-1.648252196	0.403113428
C	0.893547501	-0.033427485	-0.929993634
C	0.910515635	1.516661671	-0.932404133
C	1.339198976	-0.715281943	0.378916359
C	0.634895003	-0.46846648	1.569333379
H	-0.140384812	0.288282796	1.559680072
C	3.928448697	0.983608849	0.319185012
C	1.798673527	2.329338115	-0.17696972
C	3.336405421	-1.944597829	-0.73104634
C	4.054309601	0.497501029	-1.093455837
C	2.876472618	1.835838353	0.744075158
C	0.081244965	2.158314379	-1.873868575
H	-0.524868949	1.544072586	-2.528158758
C	2.887316337	2.308065107	2.071520823
H	2.077117115	2.95231071	2.399670028
C	3.490987527	-3.294068515	-1.099912312
H	2.853129478	-4.034219485	-0.626599427
C	2.654920149	-2.347397898	1.605982752
H	3.473997189	-3.059739091	1.622649373
C	4.155102502	-0.968586284	-1.364453786
C	3.890960638	1.946717773	2.97212007
H	3.867048674	2.321866112	3.990845331
C	4.923300206	0.615740506	1.243641206
H	5.717552027	-0.046313739	0.912564923
C	1.714119307	3.735823173	-0.30913893
H	2.392585473	4.346254275	0.279363166
C	0.910805339	-1.149057594	2.756370264
H	0.348950289	-0.92297545	3.658347373
C	1.920101792	-2.118494273	2.768387037
H	2.150999348	-2.667166915	3.676586733
C	4.130184437	1.385218839	-2.103634651
C	0.026693393	3.545458317	-2.01248672
H	-0.631513715	3.992915834	-2.751703328
C	4.916106098	1.091361859	2.555771592
H	5.702800008	0.79893105	3.244561392
C	5.096525999	-1.400000259	-2.319377727
H	5.745643307	-0.658340106	-2.773000429
C	4.41240738	-3.697876471	-2.068537082
H	4.495017691	-4.747222629	-2.335514117
C	0.834526628	4.348849953	-1.199485183
H	0.806720413	5.431111436	-1.28354154
C	5.227646045	-2.742620107	-2.679332953
H	5.964148994	-3.036689257	-3.421000749
H	4.098553899	2.454216593	-1.923522213
H	4.176729996	1.06416936	-3.138085832
C	-0.889463309	-0.59590348	-1.056031509
H	-1.083377725	-0.348309168	-2.101334878
H	-0.716013432	-1.671532095	-0.964790778
P	-2.588266375	-0.335663537	-0.19426311
C	-3.218707065	-1.929501729	0.561999928
C	-2.62696561	0.941062758	1.175688199
C	-3.885743822	0.231697599	-1.426269528

C	-2.320016841	-2.732143468	1.286700866
C	-4.548566869	-2.343379928	0.386146194
C	-3.203241685	0.638642278	2.418934163
C	-2.094753295	2.219853477	0.939677637
C	-3.89341475	-0.353427349	-2.703758733
C	-4.838319178	1.208146419	-1.097858429
C	-2.759211488	-3.94228765	1.834045572
H	-1.290203335	-2.419430919	1.427431794
C	-4.977443966	-3.55790624	0.934542213
H	-5.243897427	-1.729995796	-0.176060277
C	-3.242378991	1.612233699	3.424556458
H	-3.613126053	-0.346891758	2.607276802
C	-2.138005266	3.186780202	1.949804936
H	-1.632193868	2.458505622	-0.010629564
C	-4.850954871	0.037931641	-3.645139624
H	-3.154764055	-1.104246819	-2.965605899
C	-5.792944691	1.597219948	-2.045471281
H	-4.833068495	1.668740168	-0.11654894
C	-4.085105234	-4.357444561	1.657991289
H	-2.062831978	-4.559168068	2.392172915
H	-6.005183835	-3.876777224	0.794142044
C	-2.70996718	2.885198974	3.19186932
H	-3.684872847	1.372524655	4.38592512
H	-1.715180361	4.168066331	1.763260079
C	-5.800222312	1.013918625	-3.317520424
H	-4.850371892	-0.414071989	-4.631348286
H	-6.524941799	2.35621568	-1.78939829
H	-4.419644462	-5.299746855	2.079555356
H	-2.737289241	3.636571255	3.974430558
H	-6.538758412	1.320291019	-4.051116851

Table S13. Cartesian coordinate and geometry of TS **Bathtub_CC**.

O	-3.49209997	0.549498344	2.512709421
C	-4.446431024	1.39994217	-0.300054682
C	-3.580800431	-0.191666223	1.509560594
C	-2.914530486	-1.53788379	1.51277166
C	-4.516692214	0.165005549	0.389508823
C	-5.565490836	-0.728942537	0.121174184
H	-5.600904139	-1.673432804	0.655735624
C	-2.021865826	-0.196425053	-1.232575948
C	-2.387463123	-2.172398787	0.355802677
C	-3.366579897	2.40355345	-0.072671653
C	-1.475421646	0.687407869	-0.163611914
C	-2.400808477	-1.551642807	-1.002575377
C	-2.813361298	-2.175081428	2.761537095
H	-3.200026642	-1.650913195	3.628634753
C	-2.821910606	-2.343607841	-2.088469758
H	-3.133781247	-3.366549679	-1.899287407
C	-3.763571596	3.741011712	0.110678041
H	-4.82391813	3.972546082	0.082799094
C	-5.452119937	1.684516207	-1.24398748
H	-5.388252708	2.609913298	-1.807201751
C	-1.976056616	2.066464732	-0.003329534
C	-2.901840593	-1.830530921	-3.384681988
H	-3.243608996	-2.460577277	-4.199914991
C	-2.128072684	0.301789836	-2.549199074
H	-1.851928444	1.336204959	-2.728130114
C	-1.80893649	-3.448519966	0.508828382
H	-1.38998374	-3.935508082	-0.366837755
C	-6.56493521	-0.4170094	-0.804010481
H	-7.372714946	-1.117531152	-0.990406983
C	-6.504923556	0.799098734	-1.489338365
H	-7.263646611	1.052776621	-2.223468524
C	-0.335227888	0.263332615	0.536006655
C	-2.243981165	-3.44367351	2.888868877
H	-2.189732706	-3.922232687	3.861454328
C	-2.560621478	-0.492957548	-3.611794856
H	-2.624669096	-0.072717619	-4.611306451
C	-1.075546108	3.120382321	0.290751236
H	-0.016209051	2.896982194	0.34078267
C	-2.848216528	4.759233641	0.383742955
H	-3.190859791	5.778184613	0.532711784
C	-1.746640128	-4.087037269	1.751875522
H	-1.302015166	-5.074734136	1.830728629
C	-1.491655768	4.436763978	0.483436006
H	-0.758143511	5.208893558	0.699127764
H	-0.160061509	-0.802088508	0.623966923
H	-0.032496048	0.818741128	1.418067585
P	3.089745143	0.174199565	-0.226168437
C	1.359844959	0.607621721	-0.637504855
H	1.065815306	0.076203715	-1.546236949
H	1.279114096	1.686926904	-0.780989607
C	4.552927334	0.904270207	-1.195673176
C	5.362040832	1.914559228	-0.652829475
C	4.718344338	0.529543371	-2.541379566

C	6.340057784	2.530380849	-1.444539654
H	5.237065673	2.217841399	0.380785014
C	5.700472637	1.140952587	-3.324809492
H	4.083568068	-0.237967297	-2.974637699
C	6.513265858	2.144033395	-2.777898634
H	6.965157651	3.308080539	-1.017124962
H	5.829057964	0.839710266	-4.359567128
H	7.27132969	2.622589979	-3.38933333
C	3.420255918	0.679793884	1.548169756
C	4.423591098	0.040893804	2.293063328
C	2.659085662	1.702112795	2.134420865
C	4.664593554	0.426256605	3.616304954
H	5.005842465	-0.761839388	1.853232395
C	2.906740072	2.087440409	3.457154095
H	1.870466088	2.187914773	1.57256893
C	3.908038649	1.450236063	4.198867586
H	5.435938578	-0.076259521	4.190832592
H	2.311747924	2.87564352	3.906040061
H	4.093334215	1.745090437	5.226552612
C	3.295120721	-1.673812637	-0.373696939
C	2.167797809	-2.506552638	-0.290366631
C	4.571231741	-2.240800675	-0.536150506
C	2.318678348	-3.895097982	-0.371700848
H	1.180857845	-2.079901522	-0.16759108
C	4.714379412	-3.630248237	-0.614177118
H	5.445522882	-1.604509996	-0.619711936
C	3.589281217	-4.459394687	-0.531333987
H	1.44147058	-4.529902847	-0.306599368
H	5.701733042	-4.061704437	-0.743145897
H	3.702679386	-5.536889076	-0.592381884

7. Others

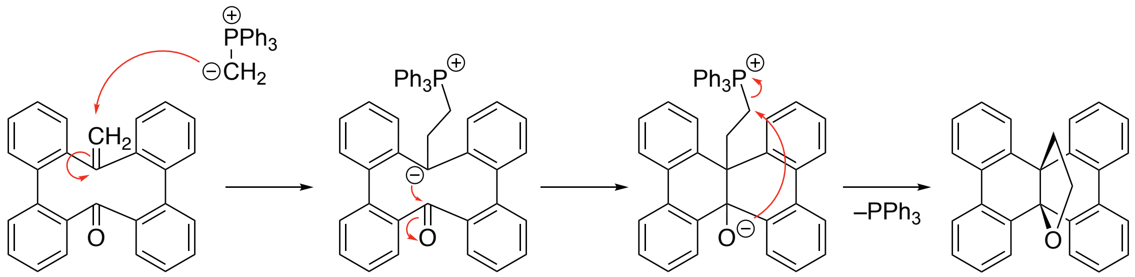

Figure S21. Proposed mechanism for the generation of **4** from **3**.

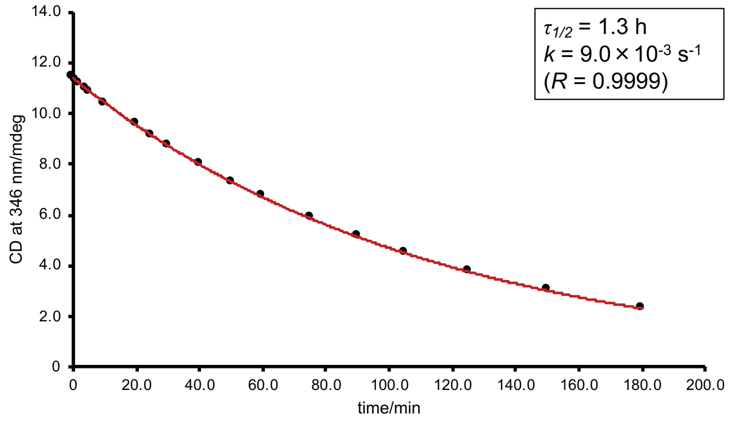

Figure S22. Plots of absolute values of CD intensities of **1** at 346 nm versus time in *o*-dichlorobenzene at 170 °C.

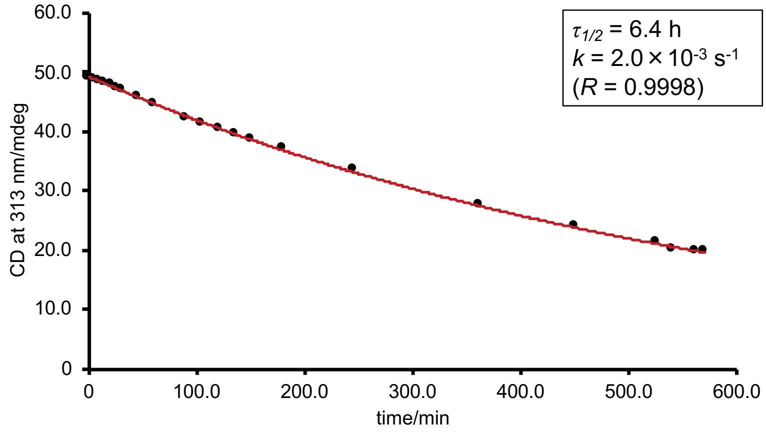

Figure S23. Plots of absolute values of CD intensities of **3** at 313 nm versus time in *o*-dichlorobenzene at 170 °C.

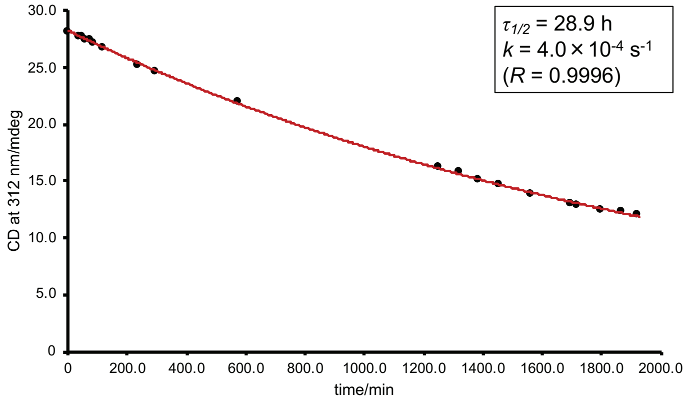

Figure S24. Plots of absolute values of CD intensities of **5** at 312 nm versus time in *o*-dichlorobenzene at 170 °C.

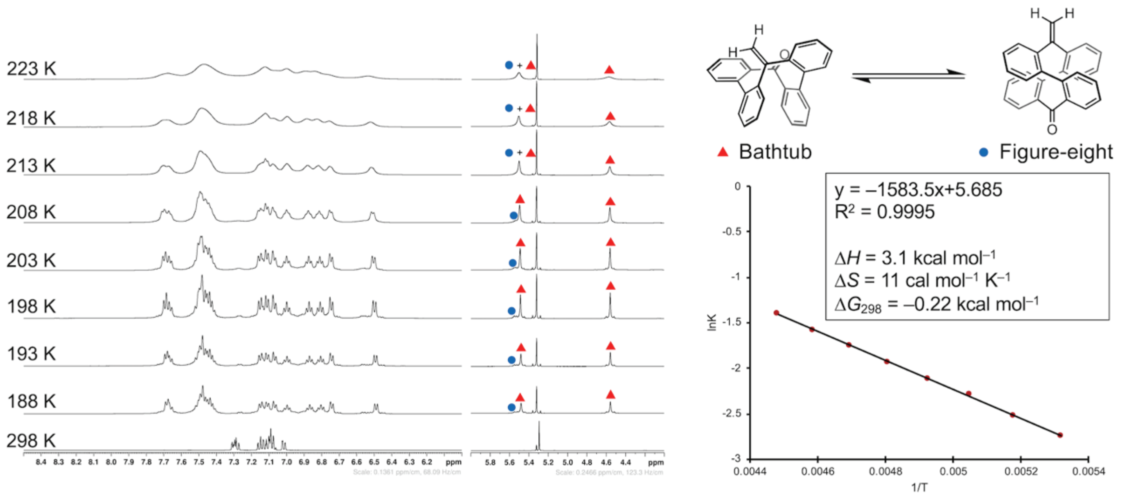

Figure S25. (a) VT ^{1}H NMR spectra of **3** in CD_2Cl_2. (b) van't Hoff plot.

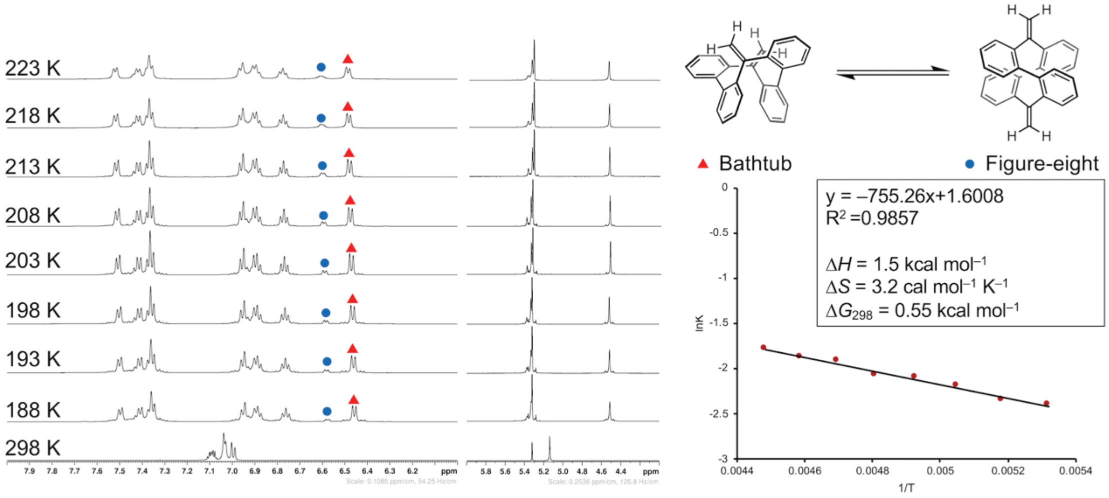

Figure S26. (a) VT ^{1}H NMR spectra of **5** in CD_2Cl_2. (b) van't Hoff plot.

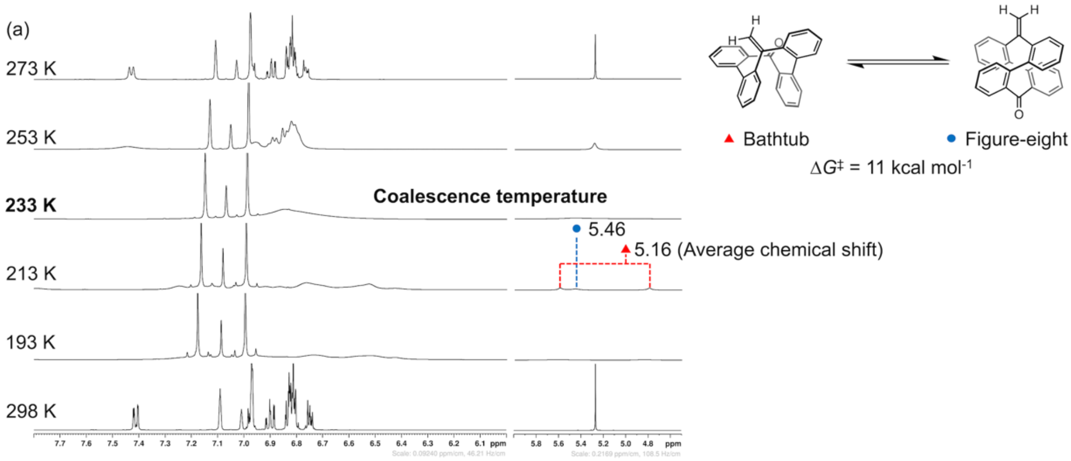

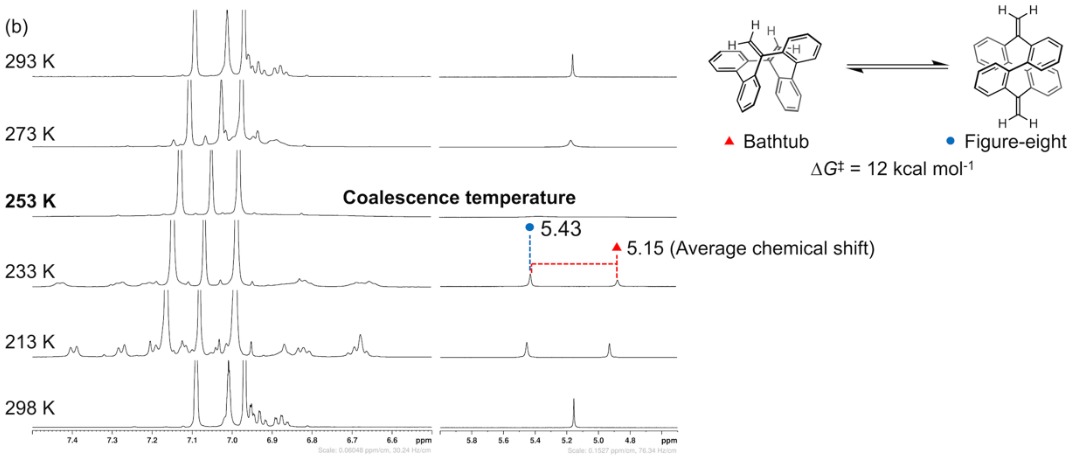

Figure S27. VT ^{1}H NMR spectra of (a) **3** and (b) **5** in toluene-d_8. Activation barriers were estimated according to the literature.[6]

8. References

1. (a) Suszko, S.; Schillak, R. *Roczniki Chem.* **1934**, *14*, 1216. (b) Yoshina, R.; Hirano, J.; Nishimoto, E.; Sakamoto, Y.; Tajima, K.; Minabe, S.-s.; Uyanik, M.; Ishihara, K.; Ikai, T.; Yashima, E.; Omine, T.; Ishiwari, F.; Saeki, A.; Kim, J.; Oh, J.; Kim, D.; Liu, G.; Yasuda, T.; Shinokubo, H.; Fukui, N. *J. Am. Chem. Soc.* **2024**, *146*, 29383.
2. Hussain, M. I.; Feng, Y.; Hu, L.; Deng, Q.; Zhang, X.; Xiong, Y. *J. Org. Chem.* **2018**, *83*, 7852.
3. Gaussian 16, Revision B.01, M. J. Frisch, G. W. Trucks, H. B. Schlegel, G. E. Scuseria, M. A. Robb, J. R. Cheeseman, G. Scalmani, V. Barone, G. A. Petersson, H. Nakatsuji, X. Li, M. Caricato, A. V. Marenich, J. Bloino, B. G. Janesko, R. Gomperts, B. Mennucci, H. P. Hratchian, J. V. Ortiz, A. F. Izmaylov, J. L. Sonnenberg, D. Williams-Young, F. Ding, F. Lipparini, F. Egidi, J. Goings, B. Peng, A. Petrone, T. Henderson, D. Ranasinghe, V. G. Zakrzewski, J. Gao, N. Rega, G. Zheng, W. Liang, M. Hada, M. Ehara, K. Toyota, R. Fukuda, J. Hasegawa, M. Ishida, T. Nakajima, Y. Honda, O. Kitao, H. Nakai, T. Vreven, K. Throssell, J. A. Montgomery, Jr., J. E. Peralta, F. Ogliaro, M. J. Bearpark, J. J. Heyd, E. N. Brothers, K. N. Kudin, V. N. Staroverov, T. A. Keith, R. Kobayashi, J. Normand, K. Raghavachari, A. P. Rendell, J. C. Burant, S. S. Iyengar, J. Tomasi, M. Cossi, J. M. Millam, M. Klene, C. Adamo, R. Cammi, J. W. Ochterski, R. L. Martin, K. Morokuma, O. Farkas, J. B. Foresman, and D. J. Fox, Gaussian, Inc., Wallingford CT, **2016**.
4. (a) Becke, A. D. *Phys. Rev. A* **1988**, *38*, 3098. (b) Lee, C.; Yang, W.; Parr, R. G. *Phys. Rev. B* **1988**, *37*, 785.
5. a) Maeda, S.; Harabuchi, Y.; Sumiya, Y.; Takagi, M.; Suzuki, K.; Hatanaka, M.; Osada, Y.; Taketsugu, T.; Morokuma, K.; Ohno, K. GRRM17, see http://iqce.jp/GRRM/index_e.shtml (accessed date: 21th May, 2025); b) Maeda, S.; Ohno, K.; Morokuma, K. *Phys. Chem. Chem. Phys*. **2013**, *15*, 3683-3701.
6. Gasparro, F. P.; Kolodny, N. H. *J. Chem. Educ.* **1977**, *54*, 258.
